# The Fine Tuning of Pain Thresholds: A Sophisticated Double Alarm System

**DOI:** 10.1371/journal.pone.0010269

**Published:** 2010-04-23

**Authors:** Léon Plaghki, Céline Decruynaere, Paul Van Dooren, Daniel Le Bars

**Affiliations:** 1 Unité READ, Université catholique de Louvain, Brussels, Belgium; 2 CESAME, Université catholique de Louvain, Louvain-La-Neuve, Belgium; 3 Team “Pain”, INSERM UMRS 975, CNRS UMR 7225, Paris, France; 4 Université Pierre et Marie Curie, Faculté de Médecine UPMC, Paris, France; University of Sydney, Australia

## Abstract

Two distinctive features characterize the way in which sensations including pain, are evoked by heat: (1) a thermal stimulus is always progressive; (2) a painful stimulus activates two different types of nociceptors, connected to peripheral afferent fibers with medium and slow conduction velocities, namely Aδ- and C-fibers. In the light of a recent study in the rat, our objective was to develop an experimental paradigm in humans, based on the joint analysis of the stimulus and the response of the subject, to measure the thermal thresholds and latencies of pain elicited by Aδ- and C-fibers. For comparison, the same approach was applied to the sensation of warmth elicited by thermoreceptors. A CO_2_ laser beam raised the temperature of the skin filmed by an infrared camera. The subject stopped the beam when he/she perceived pain. The thermal images were analyzed to provide four variables: true thresholds and latencies of pain triggered by heat via Aδ- and C-fibers. The psychophysical threshold of pain triggered by Aδ-fibers was always higher (2.5–3°C) than that triggered by C-fibers. The initial skin temperature did not influence these thresholds. The mean conduction velocities of the corresponding fibers were 13 and 0.8 m/s, respectively. The triggering of pain either by C- or by Aδ-fibers was piloted by several factors including the low/high rate of stimulation, the low/high base temperature of the skin, the short/long peripheral nerve path and some pharmacological manipulations (e.g. Capsaicin). Warming a large skin area increased the pain thresholds. Considering the warmth detection gave a different picture: the threshold was strongly influenced by the initial skin temperature and the subjects detected an average variation of 2.7°C, whatever the initial temperature. This is the first time that thresholds and latencies for pain elicited by both Aδ- and C-fibers from a given body region have been measured in the same experimental run. Such an approach illustrates the role of nociception as a “double level” and “double release” alarm system based on level detectors. By contrast, warmth detection was found to be based on difference detectors. It is hypothesized that pain results from a CNS build-up process resulting from population coding and strongly influenced by the background temperatures surrounding at large the stimulation site. We propose an alternative solution to the conventional methods that only measure a single “threshold of pain”, without knowing which of the two systems is involved.

## Introduction

Pain is an alarm system that protects individual organisms from potential or actual physical threats. The “natural experiments” provided by pathological cases of congenital insensitivity to pain illustrate clearly that pain abolition is a negative factor for survival: without a protective environment, these patients, perpetually affected by burns, wounds and fractures, have a very short life expectancy [1]. The Darwinian perspective suggests that the physiological system that produces the complex sensation of pain in mammals evolved through selection stages to their profit [2], [3]. We can even imagine a pre-eminent role for pain during the evolution of higher species because natural selection is based mainly on the premature death of the weakest or least protected individuals, whether during conflicts with other members of their species, attacks by predators, accidents or other reasons. The most endowed individuals protect themselves from such “painful” - i.e. dangerous and/or threatening - situations and therefore will survive more consistently. This requires a more efficacious system based on more sophisticated mechanisms for full accomplishment of both the biological and the psychophysical requirements for detection of, and reaction to, physical menaces. One of these threats is heat because life is possible only in a narrow range of body temperatures (e.g. 35–41°C in humans).

At the molecular level, a large number of transient receptor potential cation channels evolved from an early stage for signaling temperature changes in these ranges [4]–[7]. At a whole body level in mammals, the skin can be considered as a sense organ, which unflaggingly “sounds” the interface between the external environment and the internal milieu. Heat injury to the skin is usually modeled as a biophysical process based on the Arrhenius chemical reaction rate equation [8]. Either experimentally or in natural life, these processes are confronted with a broad range of exposure times and temperatures, two inversely related prime determinants of thermal damage: the higher the temperature the shorter the exposure time that is needed to produce a given amount of damage. The appropriate behavioral reaction to such a broad spectrum of time and temperature domains requires a reliable sensory system capable of detecting transient and sustained changes in skin temperature. To be engaged in appropriate protective reactions, the efficacy of the transient heat detectors is critically dependent on the speed of information transfer and processing time.

In the light of a recent approach developed in the rat [9], our objective was to propose an original method in humans, based on the joint use of a CO_2_ laser stimulator and infrared imaging, to study the sensations elicited by warming or heating the skin. The CO_2_ laser stimulator raises the temperature of the skin and the infrared camera records this heating. The temporal and spatial profile of the calorific power of the laser beam is known precisely [10]: its absorbance is quasi-total whatever the degree of pigmentation of the skin and the incidence of the radiation. The skin has low transparency so that the calorific energy absorbed at the level of the cutaneous surface propagates towards nerve endings sensitive to the thermal variations, which are localized just above the dermo-epidermal junction.

We developed the concept of a joint analysis of the stimulus and the response of the subject. The laser beam is applied to the skin and stopped by the withdrawal of the subject. The power varies from one stimulus to the next in a predetermined range. Their order and delay of application are randomly assigned in order to prevent any conscious or unconscious psychological bias (training, simulation, …). Each stimulation is analyzed *a posteriori* in terms of the evolution of the thermal image of the skin. The mathematical processing of these data provides numerical values of four variables characterizing a given body territory: true thresholds and latencies of pain triggered by heat via Aδ- (“first pain”) and C-fibers (“second pain”). Such an approach had never previously been considered and the more usual methods provide measurement of only a single “threshold of pain”, without knowing which of the two systems is involved. It is usually impossible to measure the latency, which prohibits any calculation of the conduction velocity of Aδ- and C-fibers. In other words, the method in development is the only one able to detect any anomalies of one and/or the other component of the nociceptive somesthesic system.

We show here that one can determine, within a single experimental session made at constant baseline temperature of the skin T_0_, the thresholds and the latencies for pain elicited by Aδ- and C-fibers. The conduction velocity of these fibers can be measured. The nature of pain, either evoked by Aδ- or by C-fibers, depends on the stimulus strength. For a given range of stimulus intensities, the partitioning of the two types of pain differs depending on the site of stimulation, with pain triggered by Aδ- fibers being favored from the distal site. The double pain phenomenon has been known since the nineteen-thirties [11]. We show here that Nature, as a good engineer, selected a redundant “double level” and “double release” alarm system. If the physical aggression occurs slowly with a gradual warming-up (e.g. staying out in the sun), the system reacts slowly but at relatively low temperatures. If the physical aggression occurs quickly, the system is much faster to react, but does so with stronger stimuli. Such an arrangement is all the more efficacious as the extremities, which are particularly vulnerable, are involved.

### Theoretical considerations: modeling the withdrawal reaction to a nociceptive heat stimulus

**Figure 1:**
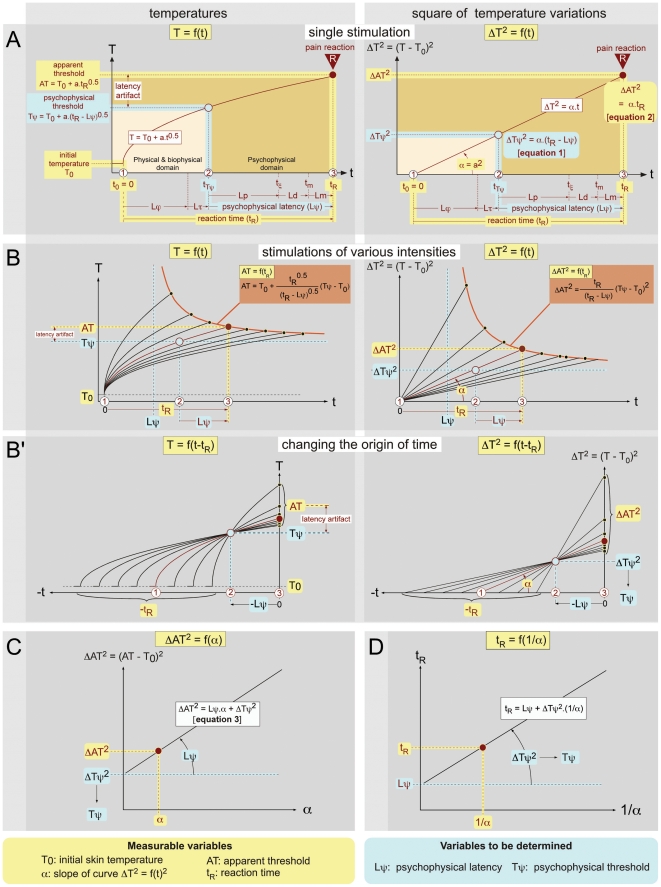
Theoretical analysis of nociceptive responses to heating. In this and the forthcoming Figures, the measurable variables are indicated with a yellow background while variables to be determined are indicated with a blue background. Individual curves of interest are shown in brown. Symbols, Abbreviations and Units can be found in **table 1**. The psychophysical response R results mainly from a serial processing along the dedicated pathways involving successive time epochs. As proposed by Luce [12], we will reserve the term latency (L) to an unobserved hypothetical time. **- A** When skin is exposed to a constant source of radiant heat, the temperature T increases with the square root of time (left graph). It increases from the initial temperature T_0_ to the AT value, reached at the time of the reaction, according to the law of physics T = f(t) = T_0_+a_*_t^0.5^. By definition, the duration of this process is t_R_, the reaction time. Expressed in terms of squared temperature variations, this relationship becomes linear (right graph): [T(t)−T_0_)]^2^ = a^2^_*_t = α.t. The time t_R_ is organized sequentially in physical (Lϕ), biophysical (Lτ) and psychophysical (Lψ) latencies. Following a period of heating (Lϕ), heat is transduced by nociceptors into neuronal spikes (period Lτ), which in turn are transmitted toward, and received by, the CNS. Lp is the transit time for these spikes to reach the CNS. Ld is the “decision” time required by the CNS for interpreting and processing this information for an order to be sent to the motor system. Lm is the time required for a motor response to be triggered. Lϕ is completely dependent upon the heating rate and varies according to the experimental protocol. The other latencies are biological variables with Lτ≪Lp [13]–[15], Lm≪Ld [17] and Lψ = Lp+Ld+Lm. Four quantities are potentially accessible to experimental measurements: T_0_, AT, t_R_ and α. **- B** Temporal evolution of the temperature of the skin during the application of thermal stimuli of various intensities. The stimulus is applied from time 0 till the response R of the subject. If one varies the power source of radiation, a series of measures can be made, including the heating of the skin from the initial temperature T_0_ up to the apparent threshold AT (left graph). The relationship AT = f(t_R_) (green curve) is an hyperbolic function with t = Lψ and T = Tψ as vertical and horizontal asymptotes respectively. In terms of squared temperature variations (right graph), the relationships are linear and the constant term α, slope of the straight lines, can be calculated. This term reflects the density of power of the heating source. The relationship ΔAT^2^ = f(t_R_) (green curve) is an hyperbolic function with t = Lψ and ΔT^2^ = ΔTψ^2^ as vertical and horizontal asymptotes respectively. **- B**’ One can modify the representation by adjusting the time scale of each individual curve for heating to the actual moment of the reaction. Such a change of origin allows one to visualize the back timing of events and to identify on the abscissa the point −Lψ and on the ordinate Tψ (left graph), or −Lψ and ΔTψ^2^ (right graph). Note that the latency artifact (AT−Tψ) increases with the stimulus intensity. **- C** Corresponding ΔAT^2^ = f(α) relationship. The intercept and the slope of this linear function represent ΔTψ^2^ and Lψ, respectively. From ΔTψ^2^, one can easily deduct the psychophysical threshold Tψ = T_0_+(ΔTψ^2^)^0,5^. **- D** Representation of the linear function t_R_ = f(1/α) = Lψ+ΔTψ^2^_*_(1/α). The intercept of the straight line with the ordinate gives the value of the psychophysical latency Lψ.

When one withdraws the hand from an intense source of heat, the process seems instantaneous. However, while both the pain sensation and the related reaction are sudden, they are in fact the consequence of a series of events, each having its own duration. To decompose this chain, one can analyze back to the moments preceding this sensation/reaction when it is elicited experimentally by nociceptive radiant heat, i.e. during the reaction time t_R_ (Fig. 1A; note that symbols, abbreviations and units can be found in table 1). The occurrence of such a reaction means that the motor system received the order to do so at time t_m_ = t_R_−Lm, where Lm represents the motor latency. The term “latency” denotes a time, which is not directly observable [12]. This order results from a decisional process, which was triggered by the arrival (and/or the accumulation) in the Central Nervous System (CNS) of a sufficient level of nociceptive information (ξ) at time t_ξ_. The duration of this process is named decisional latency or Ld. The nociceptive information had reached the CNS through peripheral fibers, which convey the impulses with a conduction velocity V. This transfer requires a certain period of time, the peripheral latency or Lp, which is determined essentially by the distance D to be traveled, i.e. the length of the fibers which transmit the information from the stimulated area of the body to the CNS. On the whole, this means that at time t_Tψ_ = t_R_−(Lp+Ld+Lm), the amount of information generated at the level of nociceptors was sufficient to trigger the reaction at time t_R_. Such a level is reached when the stimulus achieves a threshold value that we will term the “psychophysical threshold” (Tψ). This is the threshold of activation of the network of neurons at the origin of the painful sensation. Since the activation of nociceptors is not instantaneous, one might consider a transduction time or Lτ. However, taking into account the scale of the duration of the other events envisaged here, the transduction time is very brief [13]–[15]. The time period (Lp+Ld+Lm), which separates the moment at which this threshold (t_Tψ_) is reached from the moment t_R_ of the reaction, constitutes the “psychophysical latency” of the reaction (Lψ). *A priori*, it is unknown.

**Table 1:** Symbols, abbreviations and units.

Acronyms, Abbreviations	Definitions
yellow background	related to variable measured experimentally
blue background	related to latent variable to be determined
brown	related to individual curves of interest
blue color and subscript A	in relation with a pain response triggered by Aδ-fibers
red color and subscript C	in relation with a pain response triggered by C-fibers
violet color and subscript AC	in relation with a pain response triggered by both Aδ- and C-fibers (limit case)
green color and subscript W	in relation with a response triggered by non-painful thermal variation
a	α^0.5^ (°C/s)
a_AC_	α_AC_^0.5^ (°C/s)
α	slope of the squared temperature variation = a^2^ (°C^2^/s)
α_AC_	α value that separates the domains of pain responses elicited by Aδ- from C-fibers = a_AC_^2^ (°C^2^/s)
AT	apparent threshold (°C)
AT_A_	apparent threshold of pain response triggered by Aδ-fibers (°C)
AT_C_	apparent threshold of pain response triggered by C-fibers (°C)
AT_W_	apparent threshold of warm sensation (°C)
CNS	central nervous system
D	distance between the stimulation site and the dorsal horn entry zone (mm)
ΔAT	temperature variation between the initial temperature and the apparent threshold = AT−T_0_ (°C)
ΔAT_A_	temperature variation between the initial temperature and the apparent threshold of pain triggered by Aδ-fibers = AT_A_−T_0_ (°C)
ΔAT_C_	temperature variation between the initial temperature and the apparent threshold of pain triggered by C-fibers = AT_C_−T_0_ (°C)
ΔT	temperature variation with reference to the initial temperature = T−T_0_ (°C)
ΔTψ	temperature variation between the initial temperature and the psychophysical threshold of pain (°C)
ΔTψ_A_	temperature variation between the initial temperature and the psychophysical threshold of pain triggered by Aδ-fibers (°C)
ΔTψ_C_	temperature variation between the initial temperature and the psychophysical threshold of pain triggered by AC-fibers (°C)
K	composite constant grouping together the biophysical properties of skin = 2(1−r_λ_)/ρc.(π. α)^0.5^
κ	thermal diffusivity (m^2^.s^−1^)
ξ	nociceptive information
L	latency = unobserved hypothetical time (ms)
Ld	decisional latency = time required by the CNS for interpreting and processing the nociceptive information (ms)
Lm	motor latency = time from motoneurons activation up to the shortening of the muscle (ms)
Lp	peripheral latency = transit time for spikes in primary afferent to reach the entry zone in the spinal cord = Lp_t_+Lp_c_ (ms)
Lψ	psychophysical latency = time period which separates the moment at which Tψ is reached from the actual moment of the pain response R (ms)
Lψ_A_	psychophysical latency for a pain response triggered by Aδ-fibers (ms)
Lψ_C_	psychophysical latency for a pain response triggered by C-fibers (ms)
Lψ_W_	psychophysical latency for a warm sensation (ms)
Lϕ	physical latency = duration of the skin heating process from the initial skin temperature T_0_ to trigger transduction in nociceptors (ms)
Lτ	transduction latency = time required for heat to be transduced by nociceptors into neuronal spikes (ms)
p	absorption coefficient = 20.0 mm^−1^
q	laser power (mW)
Q	density of laser power (mW/mm^2^)
QST	quantitative sensory testing
R	pain response
R_A_	pain response elicited by Aδ-fibers
R_C_	pain response elicited by C-fibers
r_10.6_	reflectivity for the wave length of radiation emitted by the CO_2_ laser = 0.78% (%)
ρc	volumetric heat capacity = 4.1868 J.cm^−3^. °C^−1^ (J.cm^−3^. °C^−1^)
S	stimulation surface area (mm^2^)
S1	First sacral level of the spinal cord
t	Time (ms)
t_ξ_	time when the CNS receives a sufficient level of nociceptive information to trigger pain (ms)
t_m_	time when the motor system receives the order to trigger the reaction (ms)
t_0_	beginning of the stimulation (ms)
t_R_	moment of the psychophysical response = reaction time (ms)
t_RA_	reaction time for pain triggered by Aδ-fibers (ms)
t_RAC_	limit reaction time identical whether the experimental pain is triggered by Aδ- or C- fibers (ms)
t_RC_	reaction time for pain triggered by C-fibers (ms)
t_RW_	reaction time for a warm sensation (ms)
t_Tψ_	moment when the flow of information generated at the level of the nociceptors is sufficient to trigger a reaction (ms)
T	skin temperature (°C)
T_a_	ambient temperature (°C)
T_0_	initial temperature (°C)
Tψ	psychophysical threshold (°C)
Tψ_A_	psychophysical threshold of pain triggered by Aδ-fibers (°C)
Tψ_C_	psychophysical threshold of pain triggered by C-fibers (°C)
Tψ_W_	psychophysical threshold of warm sensation (°C)
T_c_	core temperature (°C)
T_max_	temperature of the warmest pixel of a scene (°C)
T_0_	initial skin temperature (°C)
V	conduction velocity (m/s)
V_A_	conduction velocity of Aδ-fibers that trigger pain (ms)
V_C_	conduction velocity of C-fibers that trigger pain (ms)
V_W_	conduction velocity of fibers that trigger a warm sensation (ms)

If one continues back in time, one has finally to consider the duration of the skin heating process that, starting from the initial temperature T_0_, allows the psychophysical threshold for triggering the reaction (Tψ) to be reached. This duration, the physical latency or Lϕ, is determined by the way used to increase the temperature of the skin. As a whole, the reaction time, which separates the beginning of the stimulation from the moment of the reaction, is comprised of the sum of physical, biophysical and psychophysical latencies, namely t_R_ = Lϕ+Lτ+Lψ.

To heat-up a body, one can use an infrared source of heat. The temperature is then displaced from a given level (the initial temperature T_0_) to higher levels by a process of transformation of the electromagnetic energy into calorific energy. The increase of temperature is not proportional to time but varies with its square root. This very general law of physics is verified when one turns the radiant heat towards the skin for rather short periods of time (some seconds) by means of a CO_2_ laser stimulator with a constant power of radiation. The lateral diffusion of heat by conduction attenuates this increase in temperature. However, in our experimental conditions, this effect became significant only beyond a dozen seconds, as checked in a pilot study, and will not be taken into account here. The temperature of the target zone on the skin is best described by the following equation: T(t) = T_0_+a_*_t^0.5^ or, expressed in terms of temperature variation by T(t)−T_0_ = a_*_t^0.5^. The squaring of the two terms of the equation allows one to transform this relation to a linear equation: [T(t)−T_0_]^2^ = ΔT^2^ = a^2^_*_t = α_*_t. This property is easily verified when one uses a CO_2_ laser stimulator. The constant term a (a^2^ = the slope α of the straight lines in the right graph of figure 1A) is proportional to the intensity of radiation, expressed as density of laser power Q: a = K_*_Q.

According to Meyer et al. [16], 
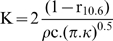


where: r_10.6_ is the reflectivity of the skin for the wave length of radiation emitted by the CO_2_ laser = 0.78,

ρc is the volumetric heat capacity and

κ is the thermal diffusivity.

In practice, for a given surface area of stimulation and a right angle of incidence of the laser beam to the skin, the term a is proportional to q, the laser power. It follows that α is proportional to q^2^. Getting control over these parameters allows one to envisage experimental paradigms whose purpose is to estimate Tψ and Lψ values, which *a priori* are unavailable.

During the stimulation period, the temperature increases to reach the psychophysical threshold of the reaction Tψ. Then, the reaction is triggered at the end of the time period Lψ, the psychophysical latency. During this time Lψ, the temperature continues to increase towards the value AT (“apparent threshold”) at the very time of the reaction. This process can be described by three key moments (Fig. 1A left):

1. the beginning of the stimulation, t = t_0_ and T = T_0_;
2. the moment of the triggering of the reaction defined by t = t_R_−Lψ and Tψ = T_0_ + a_*_(t_R_−Lψ)^0.5^;
3. the moment of the reaction defined by t = t_R_ and AT = T_0_+a_*_t_R_^0.5^.

Considering squared temperature variations, ΔT^2^ yields the following expressions (Fig. 1A right):
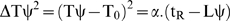
(1)and
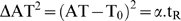
(2)


Thanks to the use of a thermographic camera, one can measure the heating of the skin from the initial temperature T_0_ up to the apparent threshold AT. The analysis of this curve allows calculation of the constant term α.

*A priori*, the following parameters can be the object of variations: (1) T_0_, the initial temperature; (2) D, the peripheral distance that the nociceptive signal must travel, which varies according to the position of the part of the body being stimulated with respect to the CNS; (3) S, the surface area stimulated; (4) q, the laser power. We chose to keep constant the first three and to vary the fourth. Variation of q means variations in the temperature rise, which one can measure. *In fine*, each test is fully summarized by four measures, namely T_0_, α, t_R_ and AT (Fig. 1B). If T_0_ remains stable during the experimental procedure, one can infer the unknowns Tψ and Lψ, which are presumably constant, from a series of trials where the power of the radiant heat source varies to produce an appropriate range of α (Fig. 1B).

### 1. Graphic determination of Tψ and Lψ by changing the time origin

One can modify the representation by adjusting the time scale of each individual curve of heating on the moment of the reaction (Fig. 1B’). Such a change of origin allows one to visualize the back timing of events and to determine the abscissa point −Lψ and ordinate point Tψ.

The equations [1] and [2] defined above can be rewritten as follows:
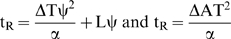
which yields
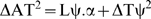
(3)


In other words, the relation ΔAT^2^ = f(α) is linear (Fig. 1C). The intercept of the straight line with the ordinate gives the value y = ΔTψ^2^ = (Tψ−T_0_)^2^. One then easily deduces the value of the psychophysical threshold: Tψ = T_0_+y^0.5^. In addition, the slope of the straight line ΔAT^2^ = f(α) gives the value of the psychophysical latency Lψ.

A second graphic representation of the data is provided in figure 1D. Since the relation t_R_ = f(1/α) is linear, the intercept of the straight line with the ordinate gives the value of the psychophysical latency Lψ. This is the theoretical latency that one would observe if the heating was instantaneous (α→∞). In addition, the slope of the straight line t_R_ = f(1/α) gives the value of (Tψ−T_0_)^2^, which then allows Tψ to be calculated.

### 2. The double afferent nociceptive system

The existence of a double afferent system, with each component having different conduction velocities and different activation thresholds, makes the problem more complex. Let us remember that nociceptive information can be conveyed by both thinly myelinated Aδ-fibers, which conduct impulses with a medium speed, and non-myelinated C-fibers, which conduct impulses more slowly (see Introduction). We will refer to V_A_ and V_C_ as the conduction velocities of Aδ- and C-fibers, which generate the nociceptive reaction. *De facto*, V_A_ is always greater than V_C_. We will refer to Tψ_A_ and Tψ_C_ as the psychophysical thresholds of the reactions triggered by Aδ- and C-fibers, respectively. These thresholds being unknown, two possibilities are offered.

1) Tψ_A_<Tψ_C_

If so, the Aδ-fibers always trigger the reaction and we are actually in the situation described above. One does observe a point of coincidence and the functions (AT−T_0_)^2^ = f(α) and t_R_ = f(1/α) are monotonic. This means that in normal conditions: (1) The CNS does not perceive as painful, information elicited by temperatures lower than Tψ_A;_ (2) The CNS perceives the nociceptive character of the temperatures that exceed Tψ_A_. There is indeed an absolute pain threshold that is determined by nociceptors connected to Aδ-fibers. This process, which requires the delay Lψ_A_ = (Lp_A_+Ld_A_+Lm), constitutes a simple alarm system. The role of C-fibers in eliciting acute pain is small, if any. The application of a brief stimulus generates the “double pain” phenomenon [17] when the temperature achieves or exceeds Tψ_C_.

2) Tψ_A_≥Tψ_C_.

**Figure 2:**
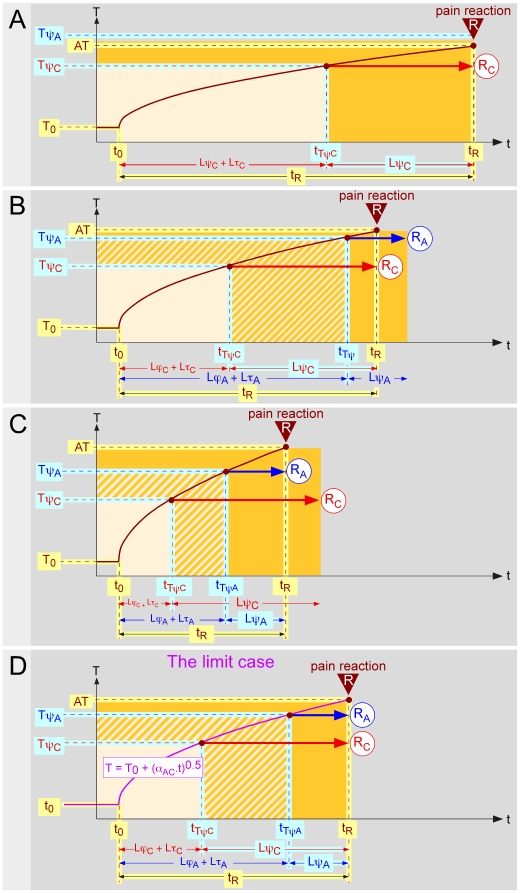
Consequences of the existence of a double afferent system (1). The correctness of the hypothesis that the pain threshold is higher when triggered by Aδ-fibers than when triggered by C-fibers means that the response is triggered first by C-fibers (red arrows) then by Aδ-fibers (blue arrows) when one progressively increases the radiant heat power. Let us refer as to R_C_ and R_A_ for these two types of responses. - **A** For the lower stimulation powers that allow the skin temperature to reach Tψ_C_ but not Tψ_A._, pain is triggered only by C-fibers following a delay Lψ_C_. - **B** There is then a small power range allowing the skin temperature to reach both Tψ_C_ and Tψ_A_, with R_C_ being triggered before R_A_. - **C** The higher stimulation powers allow the skin temperature to reach both Tψ_C_ and Tψ_A_ with pain being triggered first by the faster A-fibers following a delay Lψ_A_. This produces the classical “double pain”. - **D** The border case between situations described in B and C is characterized by pain being elicited at the very same time (R = R_C_+R_A_). This corresponds to a stimulation power characterized by a peculiar value of α we will refer as α_AC_. We will attribute the subscript “_AC_” and the purple color to the data corresponding to this particular situation.

This hypothesis is plausible if one considers data obtained during recordings of individual nerve fibers in man (Fig. 2). On average, the threshold for activation of individual Aδ-fibers is higher than that for C-fibers [15]. The practical implications of this hypothesis under normal conditions are as follows: (1) The CNS does not interpret as painful, information elicited by a temperature lower than Tψ_C;_ (2) When the temperature reaches or exceeds Tψ_C_, the CNS perceives the nociceptive character of the stimulation with a delay Lψ_C_. There is indeed an absolute pain threshold, which is determined by nociceptors connected to C-fibers; (3) When the temperature exceeds Tψ_A_, the CNS perceives the nociceptive character of the stimulation with a delay Lψ_A_. This constitutes a “double alarm system” with differential settings in terms of threshold and reaction speed. When the first set point is reached, the brain is warned but rather late. Exceeding the second set point triggers a faster warning system. The application of a very brief stimulus generates the phenomenon of “double pain” whenever the temperature exceeds Tψ_A_.

What are the consequences of the existence of a double alarm system on the experimental approach of pain by the use of a powerful radiant heat source, such as a CO_2_ laser? They are small with hypothesis (1) Tψ_A_<Tψ_C_ but considerable with hypothesis (2) Tψ_A_≥Tψ_C_ (Fig. 2). According to the latter, if one increases gradually the stimulation applied to the skin, the following situations appear successively. The stimulus does not evoke pain as long as T<Tψ_C_. When this threshold is reached, then pain is triggered by C-fibers following a delay Lψ_C_. During this period, the temperature continues to increase and several scenarios are to be considered successively. (1) Pain is triggered by the nociceptive information conveyed by C-fibers before the temperature reaches Tψ_A_ (Fig. 2A). (2) Pain is triggered by the nociceptive information conveyed by C-fibers after the temperature reaches Tψ_A_, but before the nociceptive information conveyed by Aδ-fibers has time to trigger pain (Fig. 2B). (3) Pain is triggered by the nociceptive information conveyed by Aδ-fibers before the expression of pain elicited by C-fibers (Fig. 2C).

**Figure 3:**
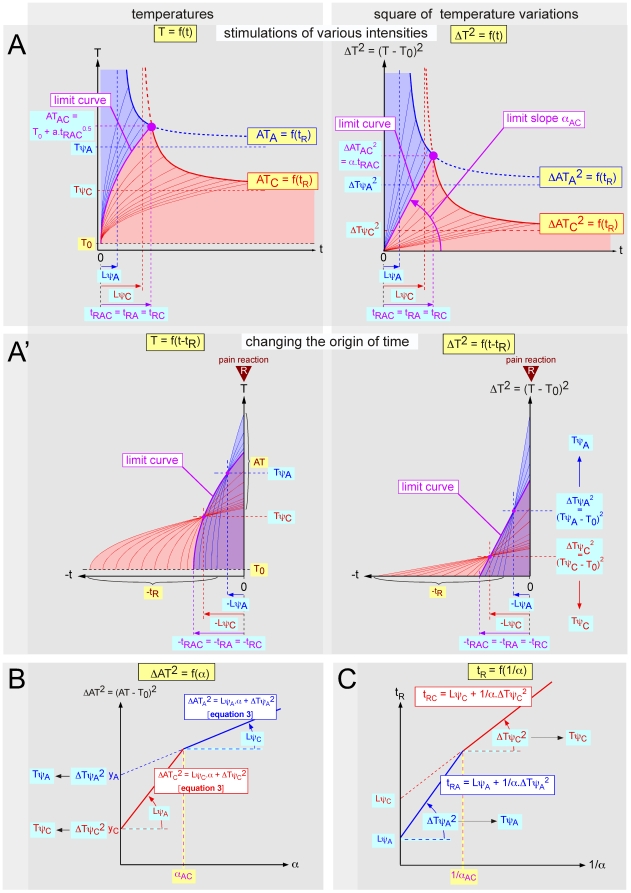
Consequences of the existence of a double afferent system (2). The correctness of the hypothesis that the pain threshold is higher when triggered by Aδ-fibers than when triggered by C-fibers has other implications. **- A** When one considers the temporal evolution of the temperature of the skin during the application of thermal stimuli of various intensities, one must take into account the two afferent systems. The relationship AT = f(t_R_) comprises two components, namely AT_A_ = f(t_RA_) and AT_C_ = f(t_RC_), respectively. These are hyperbolic functions with t = Lψ_A_ and t = Lψ_C_ as vertical asymptotes and T = Tψ_A_ and T = Tψ_C_ as horizontal asymptotes, respectively. The respective domains of heating curves that provide responses elicited by Aδ- and C-fibers are shown as blue and red areas. The two components can be seen in terms of squared temperature variations (right graph). The relationship ΔAT^2^ = f(t_R_) are hyperbolic functions with t = Lψ_A_ and t = Lψ_C_ as vertical asymptotes and T = ΔTψ_A_^2^ and T = ΔTψ_C_^2^ as horizontal asymptotes respectively. In both graphs, the transition is determined by the borderline case (“_AC_”) represented in purple, for which the reaction is triggered at the very same moment by C- and Aδ-fibers (t_RA_ = t_RC_ = t_RAC_). **- A**’ One can predict the existence of two singular points of coincidence for the heating curves when these are settled on the reaction: the points of coordinates [−Lψ_A_, Tψ_A_] and [−Lψ_C_, Tψ_C_]. When the curves are linearized by expressing the results in terms of square of differences of temperature (right graph), these curves cross each other at two points of coordinates [−Lψ_A_, ΔTψ_A_^2^] and [−Lψ_C_, ΔTψ_C_^2^]. In both graphs, the transition is determined by the borderline case shown in purple. **- B** Corresponding ΔAT^2^ = f(α) relationships. The intercepts and the slopes of these 2 linear functions represent ΔTψ_A_^2^, ΔTψ_C_^2^ and Lψ_A_, Lψ_C_, respectively. From ΔTψ_A_^2^ and ΔTψ_C_^2^, one can easily deduct the psychophysical thresholds Tψ_A_ = T_0_+(ΔTψ_A_^2^)^0,5^ and Tψ_C_ = T_0_+(ΔTψ_C_^2^)^0,5^. **- C** The corresponding t_R_ = f(1/α) relationships lead to the same values, the intercepts and the slopes of these 2 linear functions representing Lψ_A_, Lψ_C_ and ΔTψ_A_^2^, ΔTψ_C_^2^, respectively.

In the first two cases, pain is triggered by C-fibers. In the last, pain is triggered by Aδ-fibers. A “limit” case, for which the reaction began at the very same moment, whether elicited by C- or Aδ-fibers, separates these two domains of stimulation (Fig. 2D). This border case is characterized by a particular stimulation power: below, pain is triggered by C-fibers; beyond, pain is triggered by Aδ-fibers. We will attribute the subscript “_AC_” to the data corresponding to this particular situation. This situation will be represented in purple in figures, with the data corresponding to C-fibers in red and those corresponding to Aδ-fibers in blue; figure 3 extends the representation of the theoretical analysis of nociceptive responses to heating to such a situation. The equations [1] and [2] defined above can then be rewritten as follows:

Below the borderline case, pain is triggered by C-fibers:

The moment of the triggering of the reaction:
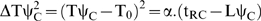
(1a)


The moment of the reaction:
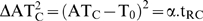
(2a)Beyond the borderline case, pain is triggered by Aδ-fibers:

The moment of the triggering of the reaction:
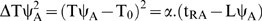
(1b)


The moment of the reaction:
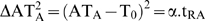
(2b)


In summary, when the heating curves are adjusted on the moment of the reaction, the theory forecasts the existence of a singular curve T = f(t), passing through two points of coincidence with coordinates [−Lψ_C_, Tψ_C_] and [−Lψ_A_, Tψ_A_], as shown in purple in figures (Fig. 3A’). If one considers the temporal evolution of the temperature of the skin during the application of increasing powers of stimulation (Fig. 3A, reading from right to left), one first records responses elicited by C-fiber activation. This continues until the limit situation and then one records responses elicited by Aδ-fiber activation. The relationship AT_A_ = f(t_R_) and AT_C_ = f(t_R_) are hyperbolic functions with t = Lψ_A_ and t = Tψ as vertical asymptotes and T = Tψ_A_ and T = Tψ_C_ as horizontal asymptotes respectively. In terms of squared temperature variations (right graph), the relationship ΔAT_A_^2^ = f(t_R_) and ΔAT_C_^2^ = f(t_R_) are hyperbolic functions with t = Lψ_A_ and t = Tψ as vertical asymptotes and ΔAT_A_^2^ = Tψ_A_ and ΔAT_C_^2^ = Tψ_C_ as horizontal asymptotes respectively. Equation 2 is written: ΔAT_AC_^2^ = α_*_t_RAC_. Adjusting the time scale of each individual curve for heating to the actual moment of the reaction and accordingly reading from left to right (Fig. 3A’), allows one to identify the two singular points with coordinates [−Lψ_A_, Tψ_A_] and [−Lψ_C_, Tψ_C_] (left graph), or [−Lψ_A_, ΔTψ_A_^2^] and [−Lψ_C_, ΔTψ_C_^2^] (right graph).

### 3. How to decide between Aδ- and C-responses

**Figure 4:**
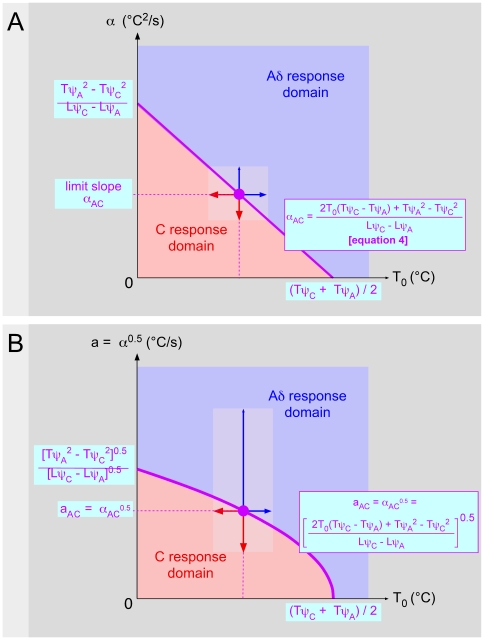
The question of Aδ- and C-fibers domains. **- A** Respective domains of responses elicited by Aδ- (blue area) and C-fibers (red area) in the plane [T_0_, α]. These domains are separated by borderline cases characterized by the fact that reaction times and apparent thresholds are identical whatever the type of fibers triggering pain. This situation links the initial temperature T_0_ and the slope α by a particular relation:
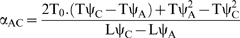
(4)For a given T_0_, there is a corresponding α_AC_ value beyond and below which, the reaction is triggered by Aδ- and C-fibers respectively (double arrow). The domain investigated in a given experimental series (dimmed area) is determined by the base temperature (here 26.8–31.6°C) and the window of used laser power (here 1.3–4.8 W) which generated a range of slope α (here 17–650°C^2^/s). **- B** Data as in A, but represented in the plane [T_0_, α^2^]. The ordinate represents the root square of α, that is a = α^0.5^. This presentation in terms of velocity of rising temperature (°C/s) is more concrete. The domain investigated in the present experiments ranged between 4.1 and 25.5°C/s.

This question means determining the limit curve between the respective domains of responses elicited by Aδ- and C-fibers. As T_0_ and α can both vary, we must determine the respective domains of the responses in the plane [T_0_, α] (Fig. 4). The borderline case is characterized by the fact that the reaction time and the apparent threshold are identical, whatever the type of fiber that triggers the experimental pain: i.e. t_RA_ = t_RC_ = t_RAC_ and AT_A_ = AT_C_ = AT_AC_. For a given T_0_ value, the response is elicited for a particular α value that we will refer to as α_AC_.

Thus, according to equations 1b and 1a, the moments of the triggering of the reactions:
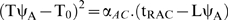


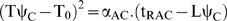
That gives the linear relation α_AC_ = f(T_0_):
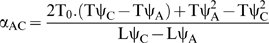
(4)


This straight line limits the domain in the plane [T_0_, α] where the responses are elicited by Aδ-fibers from the domain where they are elicited by C-fibers. For a given T_0_ value, there is an α_AC_ value beyond and below which the reactions are triggered by Aδ- and C-fibers respectively. For a given range of α values (softened area), which corresponds to a given range of power of the radiant heat source, the respective domains of responses elicited by Aδ- and C-fibers are dependent on several parameters including the distance of the stimulation site from the CNS depending on the position of the stimulated area on the body and the difference in temperature at threshold. Indeed, the relative domain of responses elicited by Aδ- versus C- fibers increases with T_0_ and decreases when the stimulation site approaches the CNS because the α_AC_ term, which is inversely proportional to (Lψ_C_−Lψ_A_), decreases.

As the term α is abstract to some extent (°C^2^/s), one can extract from it, the square root of temperature to express the data in the form of rate of rising temperature (°C/s), which is more relevant in practical terms (Fig. 4B).
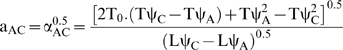



When the respective domains of Aδ- and C-responses and the limit slope α_AC_ are known, then one is able to use the approach described in figure 1C & 1D for both the Aδ- and C-responses. Indeed, the determination of the intercept of the straight line with the ordinate and the slope of the straight line ΔAT^2^ = f(α) (Fig. 3B) or t_R_ = f(1/α) (Fig. 3C) can be made separately for both types of response.

In figure 3B, the intercept of the blue straight line with the ordinate gives the value y_A_ = ΔTψ_A_^2^ = (Tψ_A_−T_0_)^2^. One can then easily derive the value of the psychophysical threshold: Tψ_A_ = T_0_+y_A_^0.5^. In addition, the slope of the straight line ΔAT_A_^2^ = f(α) gives the value of the psychophysical latency Lψ_A_. The intercept of the red straight line with the ordinate gives the value y_C_ = ΔTψ_C_^2^ = (Tψ_C_−T_0_)^2^. One can then easily derive Tψ_C_ and Lψ_C_. In figure 3C, the intercept of the red and blue straight lines t_RC_ = f(1/α) and t_RA_ = f(1/α) with the ordinate gives the values of the psychophysical latencies Lψ_C_ and Lψ_A_ respectively. The slopes of these straight lines give the value of (Tψ_C_−T_0_)^2^ and (Tψ_A_−T_0_)^2^ and the latter in turn allows the calculation of Tψ_C_ and Tψ_A_.

## Results

The present work is devoted to checking these hypotheses experimentally. The aims were as follows: (1) to verify the existence of two types of experimental pain elicited by heat, which are characterized by particular triggering properties in terms of threshold and latency; (2) to show that one can distinguish them from non-painful thermal sensations by using the same approach for comparison; (3) to demonstrate that these two types of pain are actually triggered by Aδ- and C-fibers; and (4) to verify that one can calculate both the psychophysical threshold and the psychophysical latency of these two types of pain and that one can deliberately manipulate them.

In a first step, we shall analyze an individual example and present group results from stimulation of the dorsum of the hand. In a second step, this approach will be extended to several body territories and data ensuring the differentiation of the responses elicited by Aδ-fibers from those elicited by C-fibers will be presented. In a third step, we shall show that such a differentiation can vary according to the part of the body stimulated. Finally, we shall present data concerning the influence on the responses of varying physical conditions and pharmacological manipulations.

### 1. Stimulation of the dorsum of the hand: an individual example

**Figure 5:**
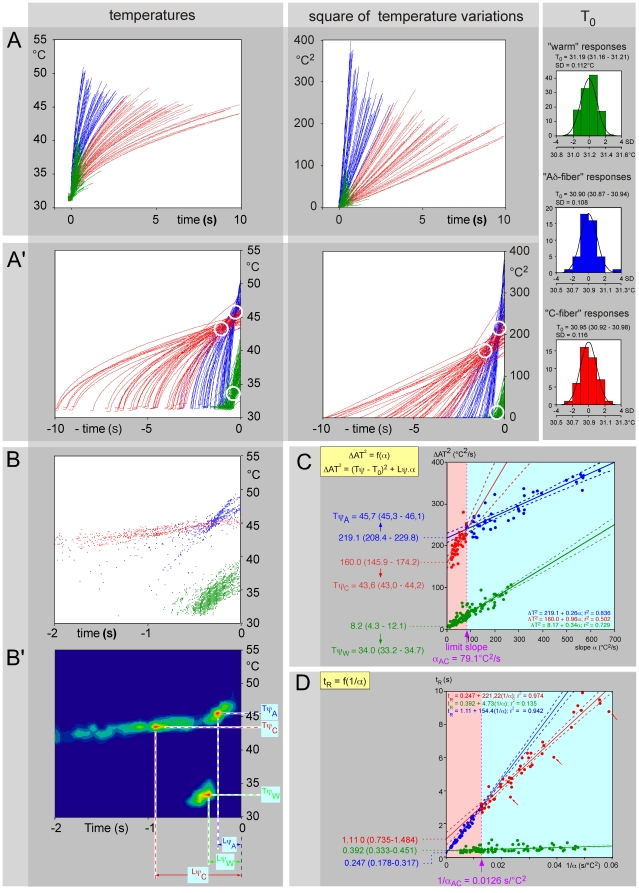
Effects of stimulation of the dorsal part of the hand in a healthy subject. For the sake of clarity, we have already attributed a color to these data on the basis of the classification defined below: blue and red for those that we believe attributable to the stimulation of nociceptive Aδ- and C-fibers, respectively (“pain test”) and green for those that we know were triggered by non-painful thermal stimulation (“warm test”). The stimulus (1–4.8 W range) was applied from time 0 until the withdrawal of the hand. **- A** Left graph: temporal evolution of the temperature of the skin recorded in the centre of the heating spot. Right graph: Identical data expressed in terms of square of the differences of temperature. All these linear relationships were highly significant and their slopes could therefore be computed confidently. The right insert shows for the three types of responses, the histograms of distribution of the initial baseline temperatures of the skin measured before the application of the laser stimuli. Each histogram is centered on the average and the theoretical normal distribution is superimposed. **- A**’ When one changes the origin to center the heating curves on the actual moment of the reaction, one can visualize the temporal evolution of the sequence of preceding events either in terms of temperature (left graph) or square of temperature variation (right graph). Note the clear tendency of these curves to cross each other in a privileged zone (open white circle). **- B** Cluster of intersections of the curves shown in A’ (left graph). **- B**’ The coordinates of the intersections of the curves shown in B were analyzed in terms of relative frequency distribution. The false colors represent the relative density of intersections. The highest probability of density of intersections was found at coordinates [−Lψ_A_ = −0.265 s; Tψ_A_ = 45.3°C], [−Lψ_C_ = −0.870 s; Tψ_C_ = 43.5°C] and [−Lψ_A_ = −0.303 s; Tψ_A_ = 33.3°C]. **- C** Calculation of the psychophysical thresholds Tψ by determination of intercepts. The figure groups together on a single graph - abscissa: slope α; ordinate: term (AT−T_0_)^2^ - all experimental points obtained with this subject during the two experimental sessions, namely the “pain” and “warm” tests. There was one and only one limit slope (α_AC_ = 79.1, marked by a vertical dotted purple line), which decided between the points of the “pain test” in two groups. The intercept of these straight lines with the ordinate gave the values of ΔTψ^2^ from which one could deduct the value of the psychophysical thresholds Tψ_A_ = 45.7°C (45.3–46.1) and Tψ_C_ = 43.6°C (43.0–44.2). The corresponding analysis of the “warm test” gave the value Tψ_W_ = 34.0°C (33.2–34.7). **- D** Calculation of the psychophysical latencies Lψ by determination of intercepts. The figure groups together all experimental points corresponding to the same experiments, but the abscissa is now the inverse of the slope α and the ordinate the reaction time t_R_. The intercept of these straight lines with the ordinate gave the values of the psychophysical latencies, Lψ_A_ = 0.247 (0.178–0.317), Lψ_C_ = 1.110 (0.735–1.484) and Lψ_W_ = 0.392 (0.333–0.451) seconds. The red arrows indicate possibilities of anticipated responses.

We present the results obtained with a 24-year-old male subject during two experimental sessions to illustrate the procedures. In one session (the “warm test”), the subject was instructed to remove his hand as soon as he perceived warmth. In the second session (the “pain test”), the instruction was to remove his hand as soon as the stimulus became painful. Figure 5A represents the individual curves for the temporal evolution of the skin temperature measured in the centre of the stimulation spot on the dorsum of his hand until active removal of the hand by the subject. The temperature increased proportionally to the square root of time, as confirmed by the rise to the square of the temperature variation (figure 5A, right graphs). Note that the initial temperature T_0_ was similar within groups of trials (figure 5A, right histograms). In each trial, the relationships [T(t)−T_0_]^2^ = f(t) were clearly linear. For clarity of presentation, we already attributed a color to these individual curves on the basis of the classification defined below. Green for those that we knew were elicited by non-painful thermal stimulation (“W” responses). Blue for those that we anticipated were related to stimulation of Aδ-nociceptors (“A” responses). Red for those that we anticipated were related to stimulation of C-nociceptors (“C” responses). By adjusting the origin of the time scale for each individual heating curve to the actual time of the reaction, one can visualize the back-timing of events (figure 5A’). Note the clear tendency of each class of curve to cross each other in a privileged zone (open white circles). Because of the stochastic nature of the psychophysical responses, the points of intersection of each curve with the others constituted a cluster in the temperature vs. time plot. These zones were determined by drawing the clusters of points of intersections (Fig. 5B) in a contour plot where the blue color represents the lowest and the red color the highest frequency of intersections with respect to the time-temperature plane (Fig. 5B’).

#### Warm test

The dorsal surface of the left hand had a base temperature of 31.2 (31.1–31.3)°C and was stimulated 92 times (Fig. 5, green curves). The relation between the square of the differential between the initial base temperature and the temperature reached at the time of the reaction ΔAT^2^, and the slope α, was described by linear regression using the least squares method and yielded the following parameters (Fig. 5C): (AT_W_−T_0_)^2^ = 8.17+0.34α (r_90_^2^ = 0.729; F_1–90_ = 242.1; p<0.001). The intercept gives the threshold of the thermal sensation [Tψ_w_ = 34.0 (33.2–34.7)°C]. The psychophysical latency was given by the constant term of the equation which expressed the reaction time t_R_ according to the inverse of the slope 1/α, t_RC_ = 0.392+4.73/α (r_90_^2^ = 0.135; p<0.001): Lψ_W_ = 0.392 (0.333–0.451) s (Fig. 5D).

#### Pain test

The dorsum of the left hand had a base temperature of 30.9 (30.8–31.0)°C and was stimulated 90 times (Fig. 5A, blue and red curves). The subject was instructed to remove his hand as soon as the stimulus became painful. Visual inspection of the cluster of points in the representation, which expresses the square of the differential between the initial temperature and the temperature reached at the time of the reaction (AT−T_0_)^2^ in function of the reaction time t_R_ (figure 5C) or the reaction time t_R_ in function of the inverse of the slope α (figure 5D), suggested that the relation presented an inflexion point. We therefore made the hypothesis that the two types of peripheral nociceptors were at the origin of this inflexion point. To estimate the value of α that separated the 90 trials into two sets, i.e. α_AC_, we solved equation 4 by using the least squares minimization procedure described in section Methods. For this particular subject, the limit slope α_AC_ = 79.1°C^2^/s. With this result and the parameters of the regression lines adjusted to the two data sets (AT_C_−T_0_)^2^ = 160+0.96 α (r_41_^2^ = 0.502; p<0.001) and (AT_A_−T_0_)^2^ = 219+0.26 α (r_45_^2^ = 0.836; p<0.001), one obtains the values of the thresholds for each set by computing the square root of the intercepts and adding T_0_, i.e. Tψ_C_ = 43.6 (43.0–44.2) and Tψ_A_ = 45.7 (45.3–46.1)°C.

On the basis of the same dichotomy, the representation which expressed the reaction time t_R_ according to the inverse of the slope 1/α yielded the corresponding psychophysical latencies by way of the equations (figure 5D): t_RC_ = 1.110+154.24/α (r_41_^2^ = 0.942; p<0.001) and t_RA_ = 0.247+221.22/α (r_45_^2^ = 0.974; p<0.001). The psychophysical latencies were given by the intercept of these equations: Lψ_C_ = 1.110 (0.735–1.484) s and Lψ_A_ = 0.247 (0.178–0.317) s.

Equation 4 defines the border between the respective domains for obtaining the “A” or “C” responses for any value of initial temperature T_0_ and slope α. In the present experiment, we investigated only a limited part of these domains, corresponding to a single base temperature (31°C) and to the 17–650°C^2^/s range of α (or in the 4.1–25.5°C/s range of a = α^0.5^). It is the stability of the base temperature T_0_ that legitimized the calculations presented above.

### 2. Stimulation of the dorsum of the hand: group analysis

**Figure 6:**
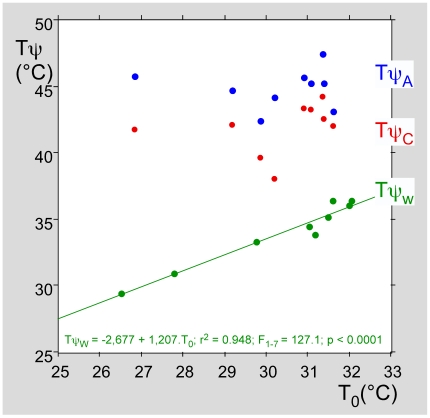
Relationships between the initial temperatures of the skin and the thresholds. Relationships between the initial temperatures of the skin and the thresholds: thermal threshold (in green), threshold of responses elicited by C- (in red) and Aδ-fibers (in blue). There was no correlation between the initial temperature of the skin and the threshold, no matter which type of fibers were triggering pain. On the other hand, the thermal threshold was very dependent on the initial temperature of the skin. A clear linear relationship was seen between the initial temperature of the skin and the threshold of heat detection (Tψ_W_ = −2,677+1,207.T_0_; r^2^ = 0.948; F_1–7_ = 127.1; p<0.0001), which means that these subjects detected variations of temperature in the 2–3°C range.

The same protocols were used in nine healthy volunteers. When the instruction was to remove the hand as soon as the stimulus became painful, it was always possible to determine a limit slope α_AC_ and, consequently, to decide between two groups of responses. These data are summarized in table 2, with the distribution of the three thresholds being presented in figure 6A. They were distributed on different windows: 29.4–36.4°C for warm, 38.1–44.3°C for pain elicited by C-fibers and 42.4–47.4°C for pain elicited by A-fibers. It is first necessary to underline a fundamental difference between the thermal and the pain thresholds as determined here. Whatever the type of fiber that triggered pain, the threshold was independent of initial skin temperature within the range obtained at normal ambient temperature (Fig. 6). In contrast, the thresholds for warmth detection were strongly influenced by the initial skin temperature. The subjects detected a variation of 2.7°C (linear relation Tψ_W_ = 2.7+1.21 T_0_; r^2^ = 0.948; F_1–7_ = 127.14; p<0.0001).

**Table 2:** Summary of psychophysical variables calculated following stimulation of the hand dorsum in nine healthy subjects.

Responses triggered by	Tψ (°C ±95% c.i.)	Lψ (ms ±95% c.i.)
Aδ-fibers	44.9 (43.9–45.8)	305 (223–387)
C-fibers	41.9 (40.7–43.2)	1018 (828–1209)
Warm fibers	34.0 (32.4–35.6)	526 (441–611)

### 3. Investigation of various body territories and conduction velocity of the fibers that trigger the sensations

**Figure 7:**
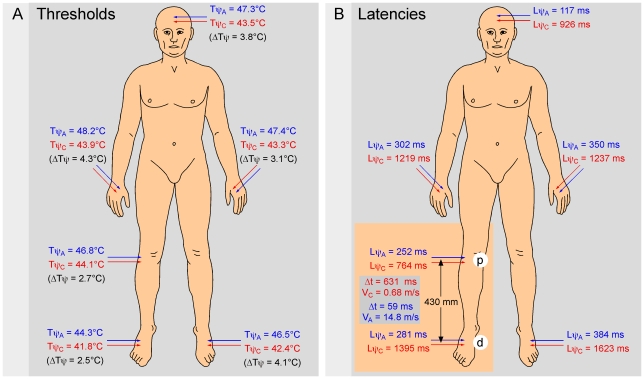
Thresholds and latencies from various territories. Six sites were investigated in this subject (front, hand, leg, foot) among which two belonged to the same dermatome on the lower limb (S1). **- A** Thresholds. The threshold Tψ_A_ of the responses triggered by Aδ-fibers was 3–4°C higher than the thresholds Tψ_C_ of the responses triggered by C-fibers. **- B** Psychophysical latencies. The latencies Lψ_A_ of the responses triggered by Aδ-fibers were several times briefer than the latencies Lψ_C_ of the responses triggered by C-fibers. The stimulation of two separate sites, the first distal on the foot (d) and the second proximal on the leg (p), allowed one to estimate the conduction velocity of the fibers responsible for the responses. The difference of the latencies of the responses attributed to Aδ-fibers was 0.059 seconds to travel the 430 mm (from d to p), which corresponds to a conduction velocity of 14.8 m/s. The difference of the latencies of the responses attributed to C-fibers was 1.395 seconds to go the same distance, which corresponds to a conduction velocity of 0.7 m/s.

Several body zones (hand, foot, leg, forehead) were investigated in four subjects. Figure 7 summarizes such an approach. In this individual example, the threshold Tψ_A_ (44.3–48.2°C) of the responses elicited by A-fibers was systematically 3–4°C higher than the threshold Tψ_C_ (41.8–44.1°C) of the responses elicited by C-fibers. These data are summarized in table 3. The stimulation of two territories in the same dermatome (S1) - one distal at the level of the foot (d) and the other proximal at the level of the leg (p) - allowed the calculation of the conduction velocities of the fibers responsible for the responses. The difference of the latencies of the responses attributed to A-fibers was 0.059 seconds to travel 0.430 m (from d to p), which corresponds to a 14.8 m/s conduction velocity. The difference of the latencies of the responses attributed to C-fibers was 0.631 seconds to cover the same distance, which corresponds to a 0.7 m/s conduction velocity.

**Table 3:** Summary of psychophysical variables calculated following stimulation of three different parts of the body, namely the forehead, the hand and the foot in four healthy subjects.

Variable	Forehead	Hand	Foot
T_0_ (°C ±95% c.i.)	32.5 (32.0–33.0)	30.5 (29.4–31.5)	29.0 (26.9–31.2)
Tψ_A_ (°C ±95% c.i.)	46.7 (46.0–47.4)	44.9 (43.5–46.3)	45.2 (43.5–47.0)
Tψ_C_ (°C ±95% c.i.)	44.3 (43.9–44.6)	42.0 (40.4–43.7)	42.4 (41.0–43.9)
Lψ _A_ (ms ±95% c.i.)	148 (102–195)	263 (217–309)	409 (319–500)
Lψ_C_ (ms ±95% c.i.)	690 (478–902)	1255 (787–1723)	1918 (1329–2506)
α_AC_ (°C^2^/s ±95% c.i.)	159.8 (101.4–218.1)	87.1 (98.0–76.1)	50.6 (39.0–62.1)

Overall, Tψ_A_ was always higher than Tψ_C_: Tψ_A_ = 45.7 (45.1–46.3)°C and Tψ_C_ = 43.0 (42.5–43.6) [t_36_ = 14.6; p<000.1] with a mean difference of 2.6 (2.3–3)°C [initial temperature = 30.4 (29.9–30.9)°C]. The mean conduction velocities of fibers responsible for the triggering of the pain responses were calculated on the basis of the results obtained in six healthy subjects. The base temperatures T_0_ and thresholds (Tψ_A_, Tψ_C_) were close, but latencies were very significantly different: 0.222 (0.164–0.280) vs. 0.264 (0.218–0.310) s for Lψ_A_ (t_5_ = 6.04; p<0.002) and 0.608 (0.354–0.861) vs. 1.333 (0.952–1.714) s for Lψ_C_ (t_5_ = 5.11; p<0.004). Overall, the average conduction velocities of both types of responses were 13.0 (10.0–15.9) and 0.8 (0.5–1.1) m/s, respectively. One therefore demonstrates here that these responses, until now referred as “A” and “C”, were effectively elicited by Aδ- and C-fibers.

### 4. Variation of the limit slope α_AC_ on the body surface

**Figure 8:**
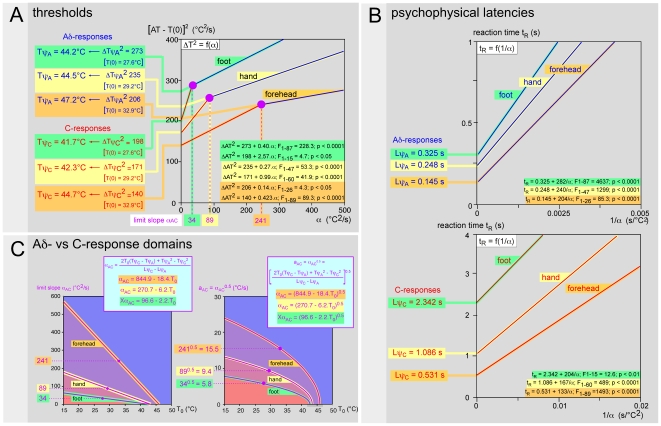
Influence of the part of the body stimulated on the limit slope α_AC_. Three physical territories, with different distances to the Central Nervous System, namely forehead, hand and foot, were stimulated. The results from an individual subject are shown. **- A** Relationship (AT−T_0_)^2^ = f(α) allowing the determination of psychophysical thresholds Tψ and limit slopes α_AC_, marked by a dotted purple line. The highest thresholds were observed from the forehead and the limit slopes α_AC_ were classified in the following order: foot < hand < forehead. **- B** Relationship t_R_ = f(1/α) allowing the determination of the psychophysical latencies Lψ_A_ (higher graph) and Lψ_C_ (lower graph). They were classified in the following order: foot > hand > forehead. **- C** Curves which delimited in the plane [T_0_, a], the domains of obtaining responses triggered by Aδ- and C-fibers respectively (see Fig. 4); the purple full circles correspond to the values determined experimentally; curves are calculated from the Tψ_C_, Tψ_A_, Lψ_C_ and Lψ_A_ values, all determined experimentally.

According to equation 4, α_AC_ is inversely proportional to (Lψ_C_−Lψ_A_), the latter being larger the further the site of stimulation is from the CNS. In other words, and all other things being equal, the α_AC_ term would seem to be lower as the site of stimulation moves in a centrifugal direction from the CNS. To verify this hypothesis, we envisaged one experimental paradigm during which three very different sites were stimulated, from the closest to the most distant from the CNS: the forehead, the hand and the foot. It was applied to four subjects with the results being qualitatively similar and we shall illustrate these observations by an individual example. In the representation which expresses the square of the differential between the initial temperature and the temperature reached at the time of the reaction (AT−T_0_)^2^ according to the slope α (figure 8A), the limit slopes α_AC_ were classified in the following order: forehead (241)>hand (89)>foot (34). In the representation which expresses the reaction time t_R_ according to the inverse of the slope α, the psychophysical latencies Lψ_A_ and Lψ_C_ (figure 8B) were indeed classified in the following inverse order: foot (0.325 and 2.342 s)>hand (0.248 and 1.086 s)>forehead (0.145 and 0.535 s), as expected.

When the function a = f(T_0_) is represented for the 3 part of the body stimulated (figure 8C) to delineate the domains for obtaining responses elicited by Aδ- or C-fiber, one notices that a_AC_ is always the weaker when the foot is stimulated and the larger when the forehead is stimulated. The plane [T_0_, a] is thus divided into the following four domains. (1) The first from which the responses were triggered by C-fibers, whatever part of the body was stimulated (red area). (2) The second from which the responses were triggered by C-fibers from the hand and forehead but by Aδ-fibers from the foot (pink area). (3) The third from which the responses were triggered by C-fibers from the forehead but by Aδ-fibers from the hand and the foot (purple area). (4) The last from which the responses were triggered by Aδ-fibers, whatever the part of the body stimulated (blue area). When one varies the initial temperature T_0_ of a given territory and applies a given range of laser powers, which generate a corresponding range of slopes α, the relative proportions of responses triggered by Aδ- and C-fibers vary. The proportion of responses elicited by Aδ-fibers increases with the initial temperature T_0_ and decreases for the benefit of responses elicited by C-fibers when this temperature falls. In summary, and for the four subjects investigated in this way (table 2), the domain of responses triggered by C-fibers is all the larger as the site of stimulation is closer to the CNS and/or the initial base temperature is low. *A contrario*, the domain of responses elicited by Aδ-fibers increases when one moves away from the CNS and/or when the initial temperature increases.

### 5. Effect of warming the skin

**Figure 9:**
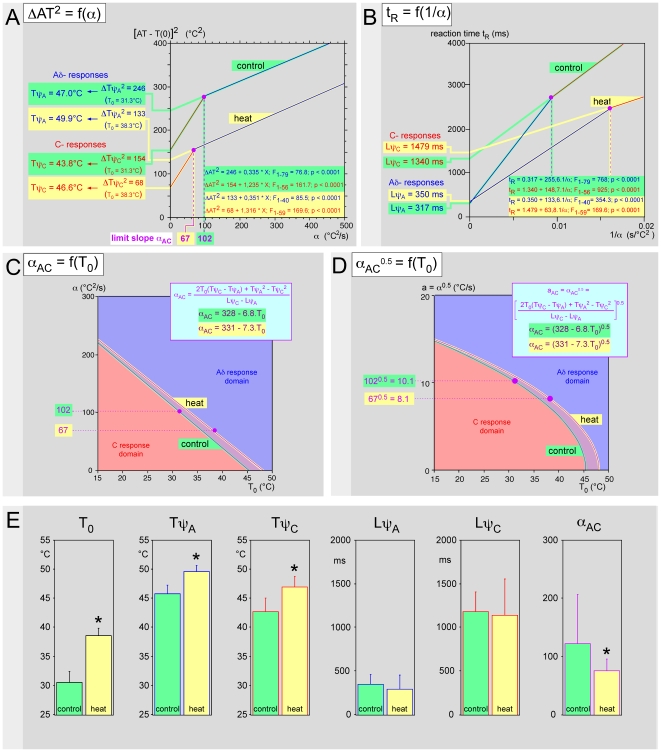
Effects of heating on the psychophysical variables. The whole dorsal surface of the hand was warmed to 38°C by means of an infrared lamp. **- A** Individual example. Relationship ΔAT^2^ = (AT−T_0_)^2^ = f(α) which allowed one to determine the psychophysical thresholds T_ψ_. A dotted purple line marks the limit slopes α_AC_ that delineate the Aδ- from the C-response domains. The shift of the straight lines ΔAT^2^ = f(α) during heating is misleading because the base temperatures were very different. The heat indeed elicited an increase of Tψ_C_ and Tψ_A_ thresholds by about 3°C. **- B** Corresponding relationship t_R_ = f(1/α) which allowed one to determine the psychophysical latencies Lψ. Note the variations of the slopes of the straight lines t_R_ = f(1/α) following heating without any modification of the intercept with the ordinate, which means that the psychophysical latencies were not modified by heating. **- C** Curves in the plane [T_0_, α] which delimit the domains for obtaining responses triggered by Aδ- and C-fibers, respectively (see Fig. 4); the purple full circles correspond to the values determined experimentally. Curves are calculated from the Tψ_C_ Tψ_A_, Lψ_C_ and Lψ_A_ values, all determined experimentally as the point of coordinates [T_0_, α_AC_]. One can see a shift of the respective domains of the responses elicited by Aδ- and C-fibers to the advantage of the latter. **- D** Identical results shown in the plane [T_0_, α_AC_^0.5^]. E. Overall mean results regarding physical (T_0_) and psychophysical (Tψ_A_, Tψ_C_, Lψ_A_, Lψ_C_ and α_AC_) variables (* = p<0.05; Wilcoxon-Mann-Whitney test).

An inverse effect was seen by increasing to 38°C the initial temperature of the dorsum of the hand by means of an infrared lamp (shut off during recordings). In the individual example of figure 9A, one sees a downward shift of the two straight lines ΔAT^2^ = f(α) and a concomitant drop of the limit slope α_AC_ following heating of the skin. However, this shift is combined with the decrease of both ΔTψ_A_^2^ and ΔTψ_C_^2^ due to the rise of thresholds elicited by the 7°C T_0_ increase. As a consequence, the decrease of ΔTψ_A_ and ΔTψ_C_ (4.2°C and 3.9°C, respectively) was less than the increase of base temperature. Calculation was necessary to verify that heat indeed elicited an increase in thresholds. One can verify on figure 9B that the corresponding psychophysical latencies were not affected, because the variations of the slopes of the straight lines t_R_ = f(1/α) were not associated with a modification of the intercepts with the ordinate. In other words, heat applied to a large surface area increases the pain thresholds elicited from a much smaller surface area (∼50 times), without concomitant variation of the corresponding psychophysical latencies. These variations were accompanied by a slight increase in the domain of the responses elicited by Aδ-fibers as shown in figures 9C & 9D where the respective domains of responses elicited by Aδ- and C-fibers are shown in the plane [T_0_, α] or the plane [T_0_, α^0.5^], respectively. Note that such changes were minimal by comparison with regards those presented in figure 8 with higher scales. The same protocol was applied to four healthy volunteers and the overall results are summarized in figure 9E. It was confirmed by non-parametric statistics (Wilcoxon-Mann-Whitney test) that both Tψ_A_ and Tψ_B_ increased significantly while the latencies did not change and α_AC_ decreased (table 4). Roughly, an average ∼8°C increase in the temperature of the hand increased both thresholds by ∼4°C.

**Table 4:** Effects on the psychophysical variables of heating the hand.

Variable	Control	Heat	Wilcoxon-Mann-Whitney test
T_0_ (°C ±95% c.i.)	30.7 (29.5–31.8)	38.7 (38.0–39.5)	P<0.05
Tψ_A_ (°C ±95% c.i.)	45.9 (45.0–46.8)	49.7 (49.1–50.4)	P<0.05
Tψ_C_ (°C ±95% c.i.)	42.8 (41.4–44.3)	47.0 (45.9–48.1)	P<0.05
Lψ_A_ (ms ±95% c.i.)	351 (281–421)	296 (194–398)	P>0.5
Lψ_C_ (ms ±95% c.i.)	1189 (1052–1325)	1147 (890–1404)	P>0.7
α_AC_ (°C^2^/s ±95% c.i.)	123.1 (70.9–175.4)	76.2 (63.4–88.9)	P<0.05

### 6. Effect of capsaicin

Finally we simply aimed to investigate a situation where a lowering of thresholds was to be observed. In two subjects (the two senior authors), the dorsum of the hand was painted with dimethylsulfoxide (DMSO) to permeate the stratum corneum, and about 1 min later with a solution of 1% capsaicin in 80% ethanol. Within minutes of the applications, the skin area painted with capsaicin showed a marked erythema and produced a spontaneous burning sensation that persisted for several hours.

**Figure 10:**
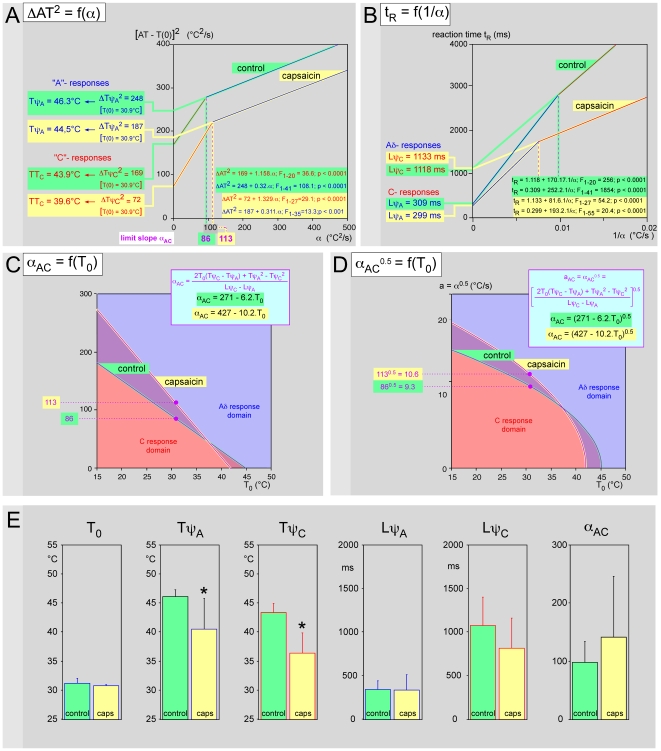
Effects of capsaicin. Presentation as in Fig. 9 (ABCD: individual example, E global data). **- A** Capsaicin elicited a downward shift of the straight lines ΔAT^2^ = (AT−T_0_)^2^ = f(α) and consequently a reduction of Tψ_A_ (−2.4°C) and Tψ_C_ (−4.9°C) thresholds because the base temperature T_0_ varied little. **- B** These effects resulted in corresponding variations of the slopes of the straight lines t_R_ = f(1/α) without modification of the intercept with the ordinate, indicating that the psychophysical latencies were not modified by the treatment. **- C** **& - D** All these modifications were responsible for a shift of the respective domains of Aδ- and C- fibers to the advantage of the former and the latter for T_0_ below and above 39°C, respectively. The white lines exemplify a hypothetical case where the sensation in triggered by Aδ-fibers in the control situation and by C-fibers following capsaicin (see discussion). **- E**. Overall mean results regarding physical (T_0_) and psychophysical (Tψ_A_, Tψ_C_, Lψ_A_, Lψ_C_ and α_AC_) variables (* = p<0.05; Wilcoxon-Mann-Whitney test).

In the individual example of figure 10A, one can see a downward shift of the two straight lines ΔAT^2^ = f(α) roughly similar to the effects seen in figure 9A. However a concomitant rise of the limit slope α_AC_ is noticeable. This shift was also combined with a decrease of both ΔTψ_A_^2^ and ΔTψ_C_^2^, but for different reasons. In this case, the main (expected) reason was the 2.4 and 4.9 fall of Tψ_A_ and Tψ_C_, respectively, while the base line T_0_ did not change. The corresponding psychophysical latencies were not affected following capsaicin application (Fig. 10B). These variations were accompanied by an increase of the domain of the responses elicited by Aδ-fibers as shown in figures 10C & 10D, at least in the normal range of skin temperatures. Interestingly the C-fiber domain increased for temperatures higher than 39°C. The significance of the expected lowering of thresholds was confirmed by the Wilcoxon-Mann-Whitney test (Fig. 10E).

## Discussion

We developed the concept of a joint analysis of the stimulus and the response of the subject for the study of sensations elicited by heat and designed experiments to check several hypotheses developed from this concept. We first verified that this approach: (1) brought to light the existence of two types of experimental pain elicited by heat, which are characterized by particular triggering properties in terms of threshold and latency; and (2) can be applied to warm sensations. We then demonstrated that the two types of pain were actually triggered by Aδ- and C-fibers, as expected. We finally verified the quality of this approach by studying the effects of a pharmacological and a physical agent, namely capsaicin and warmth, respectively. While the first gave rise to an expected effect, the second generated an original finding.

### 1. The determination of psychophysical thresholds and latencies

We showed here that, within a single experimental session made at a constant baseline temperature of the skin, one can determine the thresholds and the latencies for pain elicited by both Aδ- and C-fibers from a given body region. To the best of our knowledge, this is the first time that such information has been obtained. Indeed, the usual methods measure only a single “threshold of pain”, without knowing which of the two systems is involved and, in addition, neglect the question of the reaction time artifact [18]. This last can be visualized on figure 5A’ by considering the temperature reached at time 0, the onset of the painful feeling. Regarding the temperature reached at the very moment of the sensation/reaction for threshold introduces a systematic error, the divergence with the psychophysical (true) threshold growing with the slope of the stimulation ramp. In the present experiments, such an error reached 5–8°C and 3–5°C for pain elicited from the hand by Aδ- and C-fibers, respectively (table 5). Our observations supplement the Yarnitsky & Ochoa study [18], [19] showing that the method of limits, often used for the determination of thermal thresholds (whereby the stimulus is stopped by the subject), results in greater overestimations of threshold when the temperature rises faster. By comparison, the method of levels (where the subject's response does not influence the stimulus duration) produces identical thresholds whatever the rate of temperature variations. The notions of (“true”) behavioral and apparent thresholds developed here are fully compatible with these comments. The systematic error also increases as the stimulated sites on the skin move away from the CNS entry zone. For example it grows from the 3–5 to the 6–10°C range for pain elicited by Aδ-fibers when the stimulation site shifts from the forehead to the foot. In keeping with this statement, the pain threshold measured with the method of limits, but not the method of levels, demonstrates a gradual increase from the lowest level in the trunk to peak levels in the foot [20]. In other words, by measuring a simple/single “threshold of pain”, one introduces an overestimation of the real threshold that increases with both the rate of stimulation and the length of the peripheral neuronal path.

**Table 5:** Overall estimation of the reaction time artifact.

Site of stimulation	AT_A_−Tψ_A_ (°C ±95% c.i.)	AT_C_−Tψ_C_ (°C ±95% c.i.)
Forehead	3.9 (3.0–4.8)	3.8 (2.3–5.2)
Hand	6.6 (5.5–7.6)	4.1 (3.6–4.6)
Foot	7.4 (6.3–8.6)	4.2 (3.7–4.8)

For each individual stimulation, the difference between the (measured) apparent threshold and the (calculated) psychophysical threshold (AT−Tψ) was calculated and the maximal value of the session was taken as the larger reaction time artifact, representative of the range of variations.

The stimulus strength determined the nature of pain, evoked by either Aδ- or C-fibers. In the population of healthy volunteers studied in the present work, the psychophysical threshold of pain triggered by Aδ-fibers was always higher than the psychophysical threshold of pain triggered by C-fibers. This observation is very much in keeping with data obtained during individual peripheral fiber recordings in man and animals where average thresholds of activation were higher for Aδ- than for C-fibers [21], [22]. For example, the Aδ- and C- polymodal nociceptors (also referred as type II AMH and CMH) in hairy skin of anaesthetized monkeys had median heat thresholds of 46 and 41°C, respectively [15].

Interestingly, Yeomans and colleagues [23]–[25] studied the withdrawal of the hind paw elicited by heat in the anesthetized rat, and came to the following conclusions: a mean paw withdrawal (apparent) threshold of 47.2°C was achieved in 13.4 seconds with a low intensity lamp (heating slope 1°C/s) while the mean paw withdrawal (apparent) threshold 51.7°C was achieved in 2.6 seconds with a high intensity lamp (heating slope 6.5°C/s), suggesting that C- and Aδ- fibers were activated in the former and latter cases respectively. These observations suggest that a similar general principle governs the pain responses in rats and humans.

In humans, intense heat stimuli (step increase in skin temperature) evoke two successive pain sensations [11], [17]: the first pain, described as having a well-localized pricking quality, is of short latency and duration; the second pain, described as having a diffuse burning quality, is of longer latency and duration, outlasting the stimulus in both time and space. That a given stimulus gives rise to such different sensations - a singularity in the field of sensory physiology - is explained by the joint presence in the skin of different nociceptors, themselves connected to peripheral afferent fibers with medium and slow conduction velocities, namely Aδ- and C-fibers [26]. This singularity contributes greatly to the difficulty of studying pain because a given stimulus can activate two different sensory systems to evoke a sensation described by an identical word, namely pain. A second very important source of difficulty is the physical impossibility of heating the skin instantaneously; a thermal stimulation is always progressive. Such a situation generates a possible drawback because the subject experiences the sensation of warmth before any sensation of pain. In other words, heat is successively a conditioning and a conditioned stimulus. In some cases, notably in the case of weak stimulus intensities, anticipation was likely to have occurred in the present experiments (see Fig. 5D, red arrows). The occurrence of such anticipation following a high-speed heat ramp stimulation is probable. This is an inescapable constraint in this field.

We also provided a measurement of the latency of the psychophysical responses, a variable not usually considered. For a given body territory, the mathematical processing of the “pain test” data provides numerical values of four variables: true thresholds and latencies of pain triggered by heat via Aδ- (“first pain”) and C-fibers (“second pain”). If one considers two body territories belonging to the same metamer, distal and proximal, the conduction velocities of the fibers that triggered the sensations can then be measured. In brief, the method that we are developing allows one to appreciate with rigor the functionality of Aδ- and C-fibers as “pain triggers” in both psychophysical and physiological terms.

Finally, the threshold and latency for warm sensations could also be determined in an additional experimental session based on an identical method. To the best of our knowledge, this is again the very first time that such piece of information was provided in a single run.

### 2. The type of fiber triggering pain depends on several factors

For a given range of stimulus intensities, the partitioning of the two types of pain differs depending on the site of stimulation, with pain triggered by Aδ- fibers being favored from distal sites. A relevant example is as follows: for a given middle range power, the heat stimulus could have triggered the Aδ-evoked pain from the foot and the C-evoked pain from the leg. As one might expect, a double pain sensation is not as apparent at more proximal locations (e.g. the face) where the conduction distances are short [26].

In summary, the factors favoring the triggering of pain by either unmyelinated C-fibers or myelinated Aδ-fibers include the low/high rate of stimulation, the low/high base temperature of the skin and the short/long peripheral path. In this respect, the use of conventional sources of heating prevents ambiguous situations because the very slow rates generally used (<2°C/s) [27] predisposes the normal subject to respond to C-fibers activation before the arrival of any Aδ-evoked information within the brain. For example, with a heating slope of 1°C/s, it takes approximately five seconds to pass from the threshold of activation of C polymodal nociceptors to that of the Aδ polymodal nociceptors; this time period is ample to enable activation of C-fibers to trigger a reaction even before Aδ fibers have been activated. Whether this situation remains during the course of pathological processes, we do not know. In addition to changes in the excitability of the fibers, alterations in skin temperature might well scramble the picture, e.g. during fever and/or inflammation. The same question applies to pharmacological manipulations. Figures 10C & 10D exemplify such a hypothetical case where the sensation can be triggered by Aδ-fibers in the control situation and by C-fibers following capsaicin. Unawareness of such a possibility could lead to misinterpretations. In many instances, the variability of base skin temperature and length of peripheral path might well be the sources of apparent variability of threshold measurements.

### 3. Pain was triggered by a level detector

The present data suggest that the sensory processor scrutinized by our approach used level detectors to trigger the minimal pain sensation, here defined by the “psychophysical threshold” (Tψ). This threshold is achieved when a sufficient level of nociceptive information (ξ) baits the decisional process, following the activation of a sufficient population of individual nociceptors. Several investigators [15], [28]–[31] have observed that in the large category of thinly myelinated polymodal Aδ-nociceptors (AMH) conducting at medium velocities and unmyelinated polymodal C-nociceptors (CMH) conducting at low velocities, there are two subcategories characterized by slowly (Type I) and rapidly (Type II) adapting responses to stepped heat stimuli, respectively.

Type I-AMHs have higher threshold (>53 vs ∼46°C) and faster conduction velocities (∼25 m/s vs ∼15 m/s) than Type II-AMHs. These properties suggests that, in spite of their phasic feature, type II-AMHs were likely to be involved in the triggering of the present psychophysical responses to myelinated Aδ- fibers. Indeed the psychophysical threshold was found in the 45–47°C range, depending on body territory, and the conduction velocity of the fiber triggering the response was 13 m/s. Whether Type I AMH nociceptors also contribute to or modulate the final sensation, we do not know. Note in this respect that the Type I AMH threshold was achieved for high values of the apparent psychophysical threshold (see table 5); firing in these fibers could have therefore “colored” the sensation to the higher intensities of stimulation because of their tonic feature.

The two subcategories of CMH classes of C-nociceptive afferents exhibit similar conduction velocity (∼0.8 m/s) and radiant heat threshold (∼45 °C) [28]; both were therefore likely to be involved in the triggering of the psychophysical responses to unmyelinated C-fibers reported in the present work. Indeed the psychophysical threshold was found in the 42–45°C range, depending on body territory, and the conduction velocity of the fiber triggering the response was 0.8 m/s.

In any case, within the range of skin temperatures recorded at normal ambient temperature, the pain threshold, elicited by either Aδ- or C-fibers, was independent of initial skin temperature, a property that was not endowed by the threshold for warm sensation.

### 4. Warm sensation was triggered by a differential detector

Although we did not make a formal determination of the conduction velocity of the fibers that triggered the sensation of warmth, the psychophysical latencies of the warm responses were found to be compatible with unmyelinated C-fibers at the faster end of their range (∼2 m/s). Interestingly, there were no signs that some responses were triggered by Aδ-fibers, a finding which is keeping with direct recordings of peripheral fibers from animals and humans [32]–[36].

Hensel [37] described the threshold conditions for warm sensations from a given body territory by the following thermal parameters: (a) the absolute temperature T_0_ of the skin; (b) the rate of temperature change; and (c) the area of stimulation. In contrast to pain sensations, the absolute thresholds for warmth detection were found to be influenced strongly by the initial skin temperature: the subjects detected an average variation of 2.7°C, whatever was the initial temperature, in the 26–32°C range. Many studies of warm detection threshold have been made with the method of limits using a Peltier thermode with a 32°C base temperature. If one considers data related to the hand with a ∼10 cm^2^ area of stimulation, the detectable changes of temperature were found to be in the 1–5°C range [19]–[20], [38]–[42]. The measured warm thresholds were related directly to the rate of temperature changes, being in fact apparent thresholds as defined in the present paper; accordingly, the differences were generally attributed to the reaction time artifact [19], [39]–[40], [43]–[44]. On the other hand, the forced choice method of levels applied in similar conditions provided remarkably reproducible detectable changes of temperature in the 0.6–0.7°C range [19], [38], [40], [45]. Jamal et al. [46] refined the set-up and protocols, notably by increasing the base temperature to 34°C, the comfort zone for a naked human in a climate-controlled room, and were able to propose 0.23°C as the warm detection threshold.

It is somewhat difficult to compare these data with our present results for methodological reasons. First we did not pre-heat the target area to a base temperature. We are thus dealing with a simpler situation whereby the target area is surrounded by a larger zone at a temperature determined by both the history of the subject and the temperature of the room, free from any additional sources of stimulation. Only two temperatures are involved in the sensory processing: the initial temperature (which remains stable in the surrounding area during the test) and the temperature of the stimulated target zone. In the above-mentioned studies, three temperatures were involved in the sensory processing: the temperature of the surrounding area (that presumably remained∼constant during the test) and which was also determined by the history of the subject and the temperature of the room, the clamped base temperature and the temperature of the stimulated target zone. Nevertheless, these studies are interpreted in terms of a temporal gradient of temperature at the target site between the initial and the stimulus-elicited temperatures, neglecting the spatial gradient between the stimulated target area and the much larger surrounding skin. The additional role of the pressure by the Peltier probe is a fourth additional protagonist, which makes interpretations not so easy because of possible sensory interactions.

In our experiments, two temperatures only have to be considered: the stimulated target area and the much larger surrounding skin. The initial temperature of the target area and the temperature of the much larger surrounding skin are identical and stable during the test period; there is no ambiguity as to the gradient that triggers the sensation. In such a situation, the subjects detected an average variation of 2.7°C, a higher value than reported previously. This difference is in fact not surprising because spatial summation plays an important role in the perception of warmth: it increases with the size of the exposed surface [37], [47]–[53]. In our experiments the stimulated spot area was small (∼3 cm^2^) with a Gaussian profile, which undoubtedly means that the 2.7°C variation concerned only the 64 warmest pixels or ∼6 mm^2^. Considering the ∼two orders of magnitude difference between the surfaces used in the above-mentioned studies with thermodes and the present study with a laser beam, the ∼1/10 ratio between average detectable variations of temperature makes sense. In addition, our results are in general agreement with the fact that the rate of temperature change does not affect the detectable changes, except for very slow changing rates of less than 0.02°C/s [37], [48], [54].

By contrast with other senses, stimulation of thermal receptors depends upon a temporal thermal gradient. In the other senses, an absence of sensation is associated with near zero energy levels. Zero thermal energy would mean a −273°C skin temperature. Individual thermoreceptors have ongoing activity at static constant temperatures of 30°C or more, following a bell-shaped curve with maximum discharges at 40–43°C [55]. Within this range, warm stimuli induce an initial burst in activity in warm fibers followed by adaptation to a frequency typical for the temperature [35], while thermal sensations occur only when the stimulus temperature change occurs at above a minimal rate. It is also possible that gradients across the surface of the skin may be of some importance for signaling temperature changes. On the basis of the present data, we have no way of knowing the relative contribution of temporal and/or spatial thermal gradients to the triggering of warm sensations. In any case, we confirmed that the base temperature of the skin did not influence the thresholds for detectable variations of temperature, at least in the 26–32°C range [54]. Together, these pieces of information converge to the conclusion that a differential detector located within the CNS does trigger warm sensations.

### 5. Warming the skin increases the pain thresholds

Warming the whole hand increased both Tψ_A_ and Tψ_C_ pain thresholds without a concomitant variation in the corresponding psychophysical latencies but accompanied by a slight increase of the domain of the responses elicited by Aδ-fibers. This observation fits the recent observation in the rat of a correlation between the temperature of the tail and the behavioral threshold for withdrawal [9]. In both cases, a large warming area was superimposed over the much smaller surface area of test heating (∼two orders of magnitude). This is not a trivial effect, as the psychophysical thresholds both increased by ∼4°C following a ∼8°C increase in the temperature of the hand.

Could physical processes explain such observations? Indeed, increased warming of the skin would certainly increase perfusion of the tissue. However, the tissue volume concerned by the laser stimulus is practically confined to the thickness of the epidermis. The heat conductivity of the skin is so low and the laser stimulus so short that heat exchange by convection may be considered as negligible in our experimental conditions. This assertion was carefully checked on the recordings of the individual temperature curves. In all cases, the measured increase of skin temperature was proportional to the square root of time. The linearity of the relation [T(t)−T_0_]^2^ = α_*_t. of the heating curves was not affected by warming the hand to 38°C. The increase in pain threshold with the increased base temperature should therefore be attributed to a CNS processing phenomenon.

This finding might be interpreted as resulting from a CNS build-up process resulting from population coding. Indeed, if one considers the peripheral information emanating from the hand, one sees a huge imbalance between information from the tiny site heated by the laser (∼3 cm^2^) and the surrounding area (∼200 cm^2^). Such an imbalance is indisputably reflected in the firing of the corresponding populations of dorsal horn neurons, which means that the thermal picture of the hand received by the brain is more or less contrasted according to the base temperature. It is hypothesized that low background temperatures facilitate the detection of a nociceptive event - thus lowering the pain threshold - while higher background temperatures blur the detection of a nociceptive event - thus increasing pain thresholds. A dedicated study would seem to confirm such a view and define precisely the limits and behavior of the bandwidth through a larger range of temperatures. Interestingly, noxious-evoked discharges of neurons in the lumbar spinal cord have been reported to be inhibited by surrounding warming in both the cat and the rat [56]–[57].

To the best of our knowledge, this is the first report of such phenomenon in psychophysical terms. At first glance this appears surprising but could be explained by the technical difficulties of exploring a field where conditioned and conditioning stimuli overlap and vary in the same direction. We believe that the power of the proposed approach and methodology allowed us to overcome these difficulties. In any case, the present observations are in keeping with empirical and clinical findings. Whether moist or dry, heat has been an empirical remedy used to relieve pain. There is clinical evidence for the relief of pain by surrounding warming [58]–[60].

### 6. Interest and utility of the approach

Since current clinical electrophysiological methods that provide information regarding tactile sensations produced by rapidly-conducting, large diameter fibers, do not include the investigation of small diameter Aδ- and C-fibers, two possibilities remain. First, the study of laser evoked potentials, which is still restrained by being within the expertise of only a few laboratories in the world - the majority of which are able only to analyze the responses triggered by Aδ-fibers [61]. The second is the determination of the thermal thresholds using devices functioning according to the Peltier principle [62]–[63]. Quantitative sensory testing (QST) with thermal stimuli consisting of slow ramps of ascending (warm) or descending (cool) thermal energy delivered through a contact thermode is often considered as an important tool in assessing the function of the thermo-nociceptive system [64]–[66]. The devices are both available and affordable and the tests are easy to perform with a minimal qualification. However the main drawback is the quasi-exclusive limitation to the C-fiber functions because of the mildness of the heating process (1–5°C/s; often ≤2°C). In addition, the method necessitates the contact of the thermode with the skin (heat transfer by conduction), eliciting two problems: (1) the concomitant activation of low threshold non-nociceptive afferents which exert an inhibitory influence on pain mechanisms [67]–[68]; (2) the subordination of the rate of thermal transfer to the quality of the thermode-skin contact given by the pressure of application, a parameter which is not easy to control [18]. Possibly as a result of these inconveniences, a low level of reproducibility of these tests is often reported [64], [66], [69]–[73]. Together with the already mentioned inescapable bias of the reaction time artifact, these considerations lead one to conclude that this mode of thermal stimulation is perhaps not fully adapted to the psychophysical approach of thermal and painful sensations. A report of the American Academy of Neurology concludes with a very reserved opinion as to the utility of these methods in clinical practice [64].

We propose here an alternative solution for the study of warm/heat sensations that displays a favorable advantages/disadvantages ratio. The main advantages are: (1) stimulation without any contact of the skin; (2) simplicity of instruction and easiness of the task: (3) investigation of both Aδ- and C-fibers functions in a single testing run; (4) determination of true thresholds; (5) determination of psychophysical latencies. The main disadvantages are presently: (1) the absence of control over maximum temperature with a potential risk of burn injury; (2) the costs of a CO_2_ laser stimulator and an infrared camera; (3) the high number of stimuli required for a full completion of a test (typically 100).

The power of the laser beam makes its use potentially dangerous, in particular when the subject does not react or reacts tardily to the stimulus. Although this was not the case in our study devoted to normal subjects free from pathologies, the picture could become completely different if patients with unknown sensory capacities were to be tested. A safety system must be conceived, aimed at blocking the stimulation as soon as the temperature of the skin exceeds a predetermined precise value. Optimization and safeguarding of the method are therefore a prerequisite for further studies and are now under final development. The general objective will be to improve the quantitative sensory evaluation of the patient and, therefore, the etiologic diagnosis of his pain, in particular when it is of neuropathic origin. Note that the method can be applied to any subject whose motor faculties are not affected. In particular, it is perfectly conceivable to use it with infants, old or mentally handicapped subjects (even during sleep), provided one strictly follows the above-mentioned safety conditions.

### 7. General conclusion

**Figure 11:**
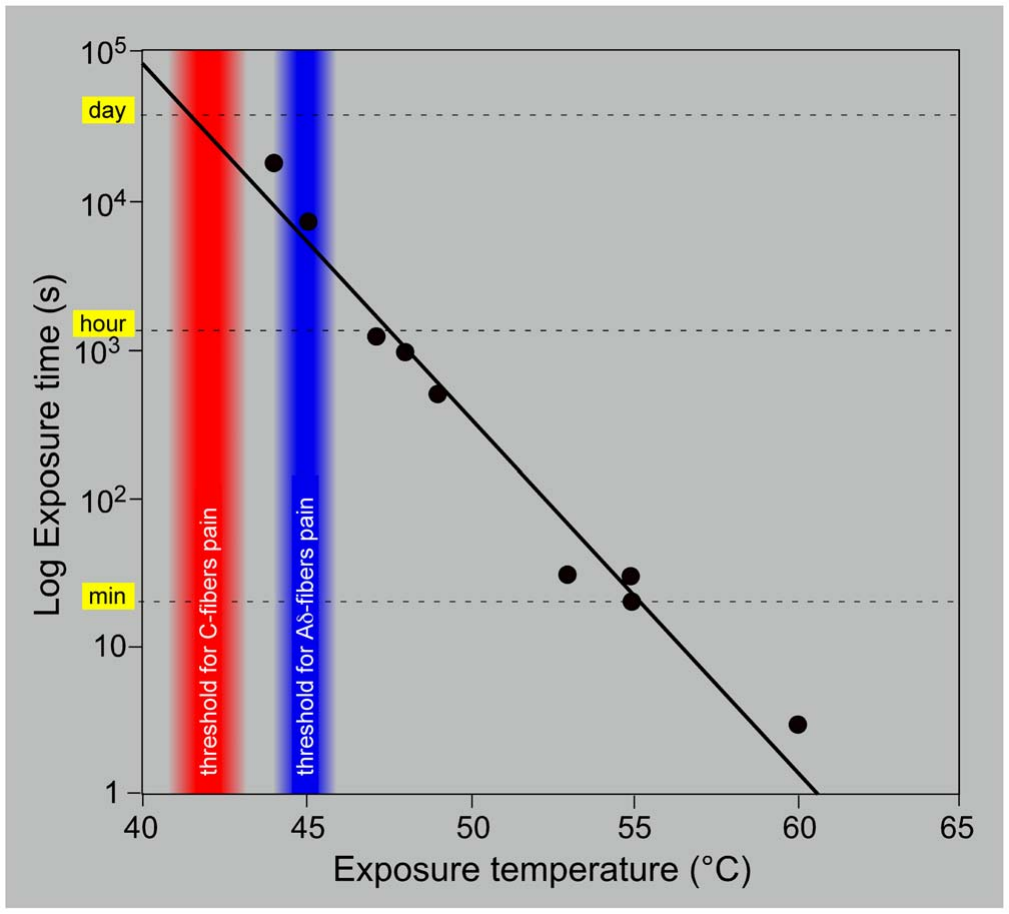
Time-surface temperature thresholds for thermal injury of Human skin. Relationship between exposure time (s) and skin surface temperature (°C) according to data from Moritz & Henriques [74]. The solid line indicates the limit between absence of a thermal lesion and a first superficial degree of skin burn (hyperemic reaction). Data related to the hand from table 2 are added as red (threshold for C-fibers pain) and blue (threshold for Aδ-fibers pain) areas.

Like a good engineer, natural selection privileged a redundant system that includes two alarms triggered at two slightly different levels. If the physical threat occurs slowly, the system is slow to react but does so at relatively low temperatures. When the physical aggression occurs quickly, the system reacts faster but only with elevated stimuli. In the latter case, the brain receives two postponed signals with two time constants, providing an automatic and inevitable temporal summation. However, if the stimulus is brief but intense, the time lag generates the phenomenon of double pain. It is remarkable that the disparity between thresholds was found to be confined to a relatively small range (2.5–3°C). Figure 11 shows the relationship between exposure time and skin surface temperature eliciting a first superficial degree of skin burn, according to data from Moritz & Henriques [74], together with the data related to the hand from table 2 as red (threshold for C-fibers pain) and blue (threshold for Aδ-fibers pain) areas. These two colored areas represent safeguard boundaries, probably promoting the learning of guarding reflexes and behaviors in normal conditions. However, such a safety margin can be strongly reduced either when the exposure time drags on (e.g. under sun light) or when the aggressive temperature reaches ten or so degrees more. Such a double mechanism thus constitutes a very effective firewall. The majority of nociceptors is activated by various types of stimulus, whether thermal, mechanical or chemical, they are called polymodal for this reason. Interestingly, many polymodal C-fiber nociceptors are activated by non-painful stimuli such as heat or a stringent vigorous friction with a massage glove. In other words, they are firing before reaching a sufficient level of nociceptive information to achieve the psychophysical threshold. Note that both receptor and psychophysical thresholds can be strongly sensitized with capsaicin treatment, as shown here. This plasticity is greatly favored by the primitive nature, poorly differentiated and totipotent, of polymodal nociceptors [75]. This is probably one of the most archaic sensory receptors, which is present even in invertebrates such as the roundworm, leech or aplysia [76]–[78]. The fact that so poorly specific nociceptors underwent the evolution of species while preserving their main characters suggests that their function is essential for the survival of individuals. If one considers the polymodal receptors as an entity, they can be conceived as a sense organ that, relentlessly, “sounds out” our whole body. In mammals, this auscultation is completed by the warm (and cold) receptors that provide additional fundamental information to the organism regarding its thermal environment for driving thermoregulatory processes that maintain a constant internal body temperature and, thereby, the basic well-being necessary for health.

We show here that the three levels of thermal sensations can be investigated rigorously on psychophysical grounds and believe that such an exploration will have important implications when patients benefit from this approach.

## Materials and Methods

### 1. Subjects

Fifteen volunteers, including 3 women (median age 33 years, range 22–57 years) participated to the study and could withdraw from it at any time, without justification. The Ethics Committee of the Université catholique de Louvain approved this study and did not make any objection from the point of view of the ethics defined by the Declaration of Helsinki. After explanation of the protocol and having obtained written informed consent, the subject was made comfortable. To stimulate the hand, he/she sat in front of a table, with a forearm in the most natural position. To stimulate the foot or the leg, the subject laid in lateral decubitus on a bed. To stimulate the forehead, the subject sat in a backwardly inclined relaxation armchair. The subjects and the experimenters wore goggles.

### 2. Materials

#### 2.1. The CO_2_ laser stimulator

**Figure 12:**
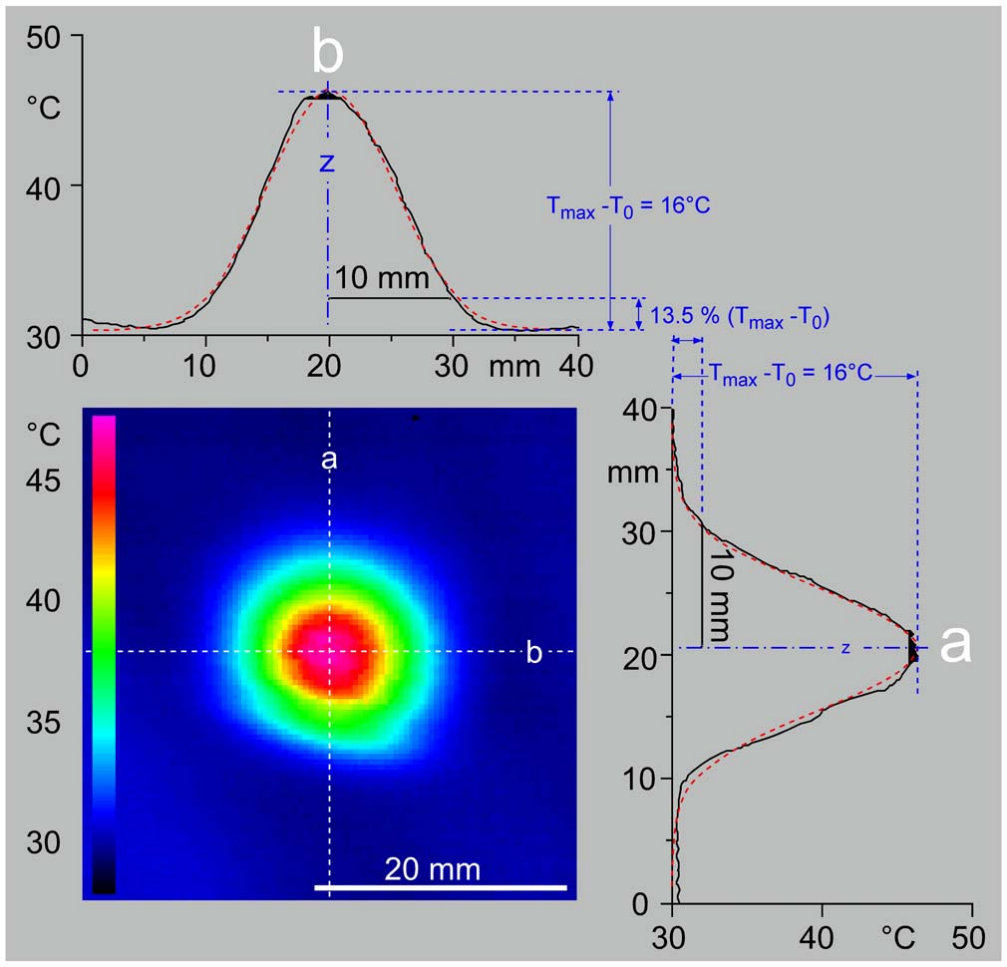
Example of thermal image of the skin recorded just before the pain response of the subject. The warmest pixel T_max_ of the scene reached 46.4°C. The spatial profiles of temperature are presented in the right (a) and above (b) the image. They correspond to the white dotted lines a and b drawn on the thermal image. The darkened zone at the top of these profiles corresponds to the 64 pixels which were used for the analysis of the temporal profiles; the average temperature of these 64 pixels is 46.1°C. The red dotted lines added to the black experimental curves are ideal Gaussian profiles. On such a picture, one can compare the radius of the stimulation spot with the radius of the laser beam. The radius of the beam is defined as the distance separating its z axis from the zone where its power was reduced to 1/e^2^ = 13.5% of its maximum. A corresponding radius of the stimulation spot resulted from these properties of the beam: the distance separating the z axis of the heating spot from the zone where the difference of temperature (T_0_−T_max_) was reduced to 13.5% of the maximum was indeed 10 mm. The related surface was ∼300 mm^2^ (∼3500 pixels).

A CO_2_ laser stimulator was used for the reasons listed below [10]. (1) It is an infra-red monochromatic radiant source with a long wavelength (10.6 µm) for which the absorbance is almost total whatever the pigmentation of the skin and the incidence of the beam. (2) Skin transparency is weak (∼100 µm), so that the calorific energy absorbed at the level of the cutaneous surface propagates towards nerve endings sensitive to the thermal variations, which are localized at the dermo-epidermal junction (60–120 µm depth). (3) The temporal and spatial profile of the radiant power is well determined. (4) It is possible to apply abrupt heating. (5) The absence of any contact with the skin allows one to avoid the concomitant activation of nerve fibers with low mechanical thresholds, which elicit tactile sensations and are sources of inhibitory processes on pain processing [67]. The surface area for stimulation was a circle determined by the Gaussian power profile of the laser beam (Fig. 12). We chose a diameter of 20 mm, for which lateral diffusion of heat by conduction was negligible below twelve seconds. Beyond this period, diffusion occurs gradually and significantly thwarts the temperature increase.

An infra-red stimulator (SIFEC, Ferrière, Belgium) built on the basis of a CO_2_ laser (maximal power 25 W; Optilas, Synrad, USA) delivered the radiant heat. A programmable impulse generator (Master 8-cp, AMPI, Israel) controlled triggering and power. A movement detector placed in front of the stimulated zone stopped the stimulation. In the absence of a withdrawal movement of the subject, the stimulus was automatically stopped at a cut-off time of 12 seconds. By construction, the stimulator continuously emitted a visible He-Ne laser beam, aligned to the CO_2_ laser beam. Thus, the zone of stimulation was monitored permanently with precision. The stimulator and the mirrors that guided the beams were positioned so that the stimulated zone was located 2.5 m from the exit window of the laser.

#### 2.2. The infrared camera

The measurement of temperature at the skin surface is justified by the convenience of use, the non-invasive character and the possibility of extrapolating the underlying temperatures by modeling. A JADE MWIR (3–5 µm) camera (CEDIP Infrared Systems, Croissy-Beaubourg, France) with a 500 µs integration time was used; this supplied images of 320×240 pixels at 25 Hz with a sensitivity of 0.02°C at 25°C. It was placed above the zone of stimulation and was run using the software Cirrus (CEDIP Infrared Systems, Croissy-Beaubourg, France). It was regularly calibrated by means of a black body BB701 (Omega Engineering, Stamford, USA), itself calibrated by means of a black body (CI SR80 CI Systems, Migdal Haemek, Israel). The software Altair (CEDIP Infrared Systems, Croissy-Beaubourg, France) allowed the monitoring of the spatial and temporal evolution of the temperature at the level of the stimulated surface area with 0.3 mm and 5.8 ms resolutions, respectively. The recording started 0.5 seconds before the laser stimulus.

### 3. Test procedure

To avoid any thermal interference and erroneous measurements of temperature, the skin of the stimulated surface should not contain hairs. The skin to be stimulated was therefore carefully depilated by means of a depilatory cream (Vichy^®^) one hour before the experiment. The temperature of the skin was the ongoing physiological temperature of the subject after acclimatization to the ambient temperature of the experimental room. For technical and scientific reasons, it was not intended to homogenize the basic temperatures, which varied according to the subject and the territories to be stimulated.

To avoid any possible visual or acoustic interference related to the triggering of the laser, the material was placed outside the visual field of the subject who wore headphones emitting white noise. When the subject was correctly installed, a first series of stimuli was applied to familiarize him or her to the experimental environment and to the sensations elicited by the stimulus. This phase was very important because it allowed the subject to establish their “implicit” response criterion. This criterion should be as close as possible to the instruction the subject received to remove the stimulated zone from the thermal radiation as early - but not before - the stimulus became painful (or that he/she perceived a temperature change in the case of the “warm test”). In particular, the subject needed to experience the sensation of warmth that precedes the sensation of pain in the case of weak stimulus intensities. About twenty stimuli were sufficient to familiarize the subject in this respect.

Since the subject had to remain perfectly motionless during a test, particular attention was paid to his or her comfort. For a given experimental condition, the experimenter took care of the constancy of the basic temperature of the stimulated skin areas during the whole session. Once this condition was fulfilled, the subject decided on the starting of the test when they felt ready. On their instruction, the experimenter engaged the generator of programmable impulse Master 8-cp, which triggered the camera and the stimulator after a random fore-period (0–5 seconds rectangular distribution). This procedure made the subject unaware of the start of stimulation. He/she withdrew actively the stimulated zone when the stimulus became painful (or detectable in the “warm test”). The withdrawal generated an impulse in the movement detector that immediately stopped the laser stimulator.

### 4. Experimental procedure (series of tests)

During one experimental session, lasting some one hundred minutes, about one hundred such stimuli were delivered. Stimulus intensities were chosen in a pseudo-random fashion in a predefined range, notably for security reasons and to take into account the stimulated zone. Indeed, for a given range of stimulus intensities, the relative proportion of responses elicited by one or the other of the two groups of fibers varied from a given territory to another, according to their distance from the CNS. The responses triggered by Aδ-fibers were favored when the territory was remote from the CNS (e.g. the foot) and the responses triggered by C-fibers were favored when the territory was close to the CNS (e.g. the forehead). It was therefore advisable to adapt the experimental protocol to these particular situations not to create a too considerable imbalance in favor of one type of responses to the detriment of the others. Following a pilot series of experiments, we applied the following CO_2_ laser stimulation powers, distributed in a range of 8 to 12 levels according to the sites: For the “pain tests”: hand 1.3–4.8 W; foot and leg 1.3–3.8 W; forehead 1.6–4.8 W. For the “warm tests” on the hand: 1.0–3.0 W. When the power range was chosen correctly, one hundred stimuli were sufficient to differentiate the responses elicited by Aδ- and C-fibers. To allow the skin to cool down back to the initial base temperature T_0_ and to avoid sensitization, the stimulation area was moved from one stimulus to the next and a minimum of five minutes separated stimuli applied to the same site. The 10–12 stimulation areas were placed in two lines orthogonal to the limb axis in order to minimize fluctuations in conduction latency.

### 5. Analysis of thermographic films

The analysis of the thermographic films comprised the following steps: (1) determination of the zone of interest in the recorded scene; (2) determination of the initial temperature T_0_ in this zone; and (3) calculation of the temporal evolution of the warmest pixels in this zone until the image preceding the movement, the ultimate point of this curve constituting the apparent threshold AT. This calculation was made by averaging the 64 warmest pixels (1 pixel = 0.41 mm by side) of each image. This choice was justified by convenience of use and by the fact that these 64 pixels ( = 10.8 mm^2^) corresponded to the top of the Gauss curve, which characterized the spatial profile of the thermal rise (Fig. 12). This procedure yielded a set of temperature curves defining the centre/maximum of the stimulation spots, elicited by a range of laser powers, including the corresponding measured values of T_0_ and AT.

The analysis of an individual temperature curve included the following steps: (1) transforming the temperature curve in a differential curve with regard to the initial temperature [T−T_0_ = f(t)]; (2) raising to the square (T−T_0_)^2^ = f(t); (3) checking the linearity of the differential curve (with excluding the initial part of the increase); and (4) recording the values of T_0_, α, (AT−T_0_)^2^. The estimation of t_R_ from the values of T_0_, AT and α was preferred to its direct measurement, because of the uncertainty regarding the exact moment of the beginning of stimulation (notably due to the sampling frequency of the camera). It sometimes happened that relationships calculated in this way were not linear. The main reason for that was the existence of slight movements of the subject, which produced breaks in the slope on the graph. The results of such trials (<10%) were discarded from further analysis.

### 6. Global analysis of data brought by a series of trials

The global analysis of the individual curves included the following steps: (1) building the initial temperature T_0_ histogram; (2) excluding trials for which T_0_ deviated from the mean with more than two standard deviations; (3) ordering the trials according to the increasing values of the slope α; (4) determining the limit slope α_AC_, which decided between responses triggered by Aδ- from those triggered by C-fibers; (5) grouping trials into two subgroups (“A” and “C”); (6) constructing the graph AT−T_0_)^2^ = f(α) for each subgroup; (7) checking the linearity of the function (AT−T_0_)^2^ = f(α) for each subgroup; (8) estimating Tψ_A_ and Tψ_C_; (9) constructing the graph t_R_ = f(1/α) for each subgroup; (10) checking the linearity of the function t_R_ = f(1/α) for each subgroup; and finally (11) estimating Lψ_A_ and Lψ_C_.

Determination of the limit slope α_AC_, which separates the responses elicited by Aδ- and C- fibers is a crucial stage and was performed as follows. The data (T_0_, AT, R, α) of all trials were ordered for increasing values of the slope α. The partitioning of the trials into two groups separated by the limit value α_AC_ of α was obtained by a least squares minimization criterion [79]. The Matlab code can be obtained from the authors on simple request.

**Figure 13:**
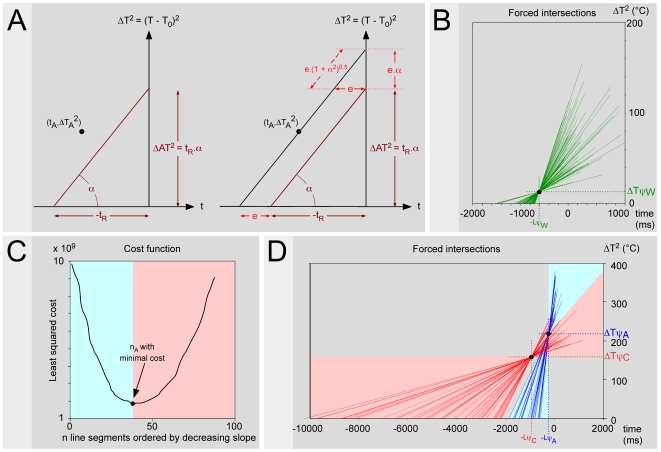
Computation of the psychophysical threshold Tψ, latency Lψ and the limit slope α_AC_ by a procedure based on a weighted least squares minimization criterion. **- A** Principle of forced intersections (see text). - B Determination of the point of forced intersections for the “warm” responses exemplified in figure 5. **- C** Least squares minimization criterion allowing to determine the limit slope α_AC_ applied to the “pain” responses exemplified in figure 5. **- D** Determination of the point of forced intersections for the A and C subgroups of psychophysical responses exemplified in figure 5.

In Figure 3A’, each line segment ΔT^2^ = f(t) corresponds to an equation t+α_i_.ΔT^2^−t_Ri_ = 0, intersecting the t-axis at t = −t_Ri_ and the ΔT^2^-axis at ΔT^2^ = t_Ri_/α_i_, which are both measured quantities. This line passes through a point (t_A_, ΔT_A_^2^) only by modifying the equation as follows t+α_i_.ΔT^2^−(t_Ri_+e_i_) = 0, where e_i_ = t_A_+α_i_.Δ_A_T^2^−t_Ri_ (Fig 13A). The minimization problem to be solved in order to force all (modified) line segments to intersect at a single point (t_A_, ΔT_A_^2^) amounts thus to the minimization of the error terms e_i_. The modified line segments intersect the t-axis at t = −(t_Ri_+e_i_) and the ΔT^2^-axis at ΔT^2^ = (t_Ri_+e_i_)α_i_ and the squared difference of the lengths of the segments is thus e_i_^2^(1+α_i_^2^). We therefore minimize the squared scaled error sum E(t_A_, ΔT_A_^2^) = Σ_i_ e_i_^2^(1+α_i_^2^) which is a weighted least squares problem in the variables (t_A_, ΔT_A_^2^) [79] (Fig 13B). A similar least squares cost is given for the line segments corresponding to another subgroup, e.g. E(t_C_, ΔT_C_^2^) for the “C” subgroup of psychophysical responses.

In order to determine the limit slope α that will settle the A and C subgroups of psychophysical responses, we minimize the weighted combination of the squared scaled error sums n_A_^2^E(t_A_,ΔT_A_^2^)+n_C_^2^E(t_C_,ΔT_C_^2^) of the two sets, where n_A_ is the number of segments corresponding to the A hypothesis and n_C_ is the number of segments corresponding to the C hypothesis (Fig 13C). Since the line segments are ordered by decreasing slope, one needs only to consider the combinations of n_A_ first segments and n_C_ = n−n_A_ last segments, with n_A_ varying from 2 to n−2 (at least 2 points are needed to solve for an intersection point). This amounts to a sequence of weighted least squares problems in the variables (t_A_, ΔT_A_^2^) and (t_C_, ΔT_C_^2^). The value n_A_ for which this cost is minimal, defines the best separation in two ordered subsets. The two subsets could then be manipulated independently (Fig 13D).

### 7. The experimental protocols

A single series of trials was generally made during a given session. When an experimental protocol required several series of tests, they were made some days apart. For the hand, two sessions were scheduled some days apart. During the “pain test”, the instruction was to remove the hand as soon as the stimulus became painful. During the “warm test”, the instruction was to remove the hand as soon as a sensation of warm was perceived. In the other cases, in particular for other body territories, only “pain tests” were performed. The power of the laser beam varied from one stimulus to the next in a predetermined range, while orders and delays of application were randomly assigned to prevent any conscious or unconscious psychophysical bias such as training or simulation. The simplicity of instruction and ease of the task were illustrated by the short period of training required in these experiments: no more than fifteen trials were needed to familiarize the subject with the task.

### 8. Measuring skin temperature

The temperature measured at the skin surface represents an approximation of the temperature reached at the level of the nociceptors, which are located at the dermo-epidermal junction, at an average depth of 100 µm [30], [80]–[81]. However, the measurement of temperature at the skin surface is justified by convenience of use, non-invasive character and possibility of computing the underlying subcutaneous temperatures by modeling. Such modeling can be found in our previous report where it is shown that the temperature reached at the dermo-epidermal junction in such experiments is very close to the measured surface temperature (see figure 10 in [9]).

For a given series of trials, the basal temperature T_0_ was stable as checked by the 95% confidence interval that was always <0.06°C.

### 9. Calculation of the conduction velocity of the fibers that triggered the reaction

Conduction velocities were calculated by stimulating two body areas situated in the same dermatome (S1), one distal on the dorsum of the foot and one proximal at the external lateral face of the superior third part of the leg. Both belong to the territory of the common peroneal nerve (dermatome S1). Knowing the distance between the two stimulation sites and the difference of psychophysical latencies would seem to provide sufficient elements for nerve conduction velocity estimations. However an additional requirement was needed: a sufficient relative number of responses elicited by Aδ- and C-fibers respectively. This number is determined mainly by the value of the limit slope α_AC_ - weak and high values favoring Aδ- and C-fiber responses respectively.

### 10. Statistical analyses

Least squares linear regressions, one-way analyses of variance (ANOVA) and Wilcoxon-Mann-Whitney test were used for statistical purposes. Statistical computations were performed with the software Staview© 5.0 (SAS Institute Inc, 1998). Other computations were made with the software Matlab© (The MathWorks, R2006a). Results were considered significant at P<0.05. Data are expressed as means ±95% confidence intervals.
